# Interventions Associated With Reduced Loneliness and Social Isolation in Older Adults: A Systematic Review and Meta-analysis

**DOI:** 10.1001/jamanetworkopen.2022.36676

**Published:** 2022-10-17

**Authors:** Peter Hoang, James A. King, Sarah Moore, Kim Moore, Krista Reich, Harman Sidhu, Chin Vern Tan, Colin Whaley, Jacqueline McMillan

**Affiliations:** 1 Division of Geriatric Medicine, Department of Medicine, University of Toronto, Toronto, Ontario, Canada; 2 Alberta Strategy for Patient Oriented Research Support Unit Data Platform, Calgary, Alberta, Canada; 3 Data and Analytics, Alberta Health Services, Edmonton, Alberta, Canada; 4 Cumming School of Medicine, University of Calgary, Calgary, Alberta, Canada; 5 Division of Geriatric Medicine, Department of Medicine, University of Calgary, Calgary, Alberta, Canada; 6 Michael G. DeGroote School of Medicine, McMaster University, Hamilton, Ontario, Canada

## Abstract

**Importance:**

Loneliness and social isolation are public health concerns faced by older adults due to physical, cognitive, and psychosocial changes that develop with aging. Loneliness and social isolation are associated with increased morbidity and mortality.

**Objective:**

To evaluate interventions, targeting older adults, associated with a reduction in loneliness and social isolation.

**Data Sources:**

OVID, CINAHL, CENTRAL, Embase, PsychINFO, Web of Science, and Scopus were searched from inception to March 2020.

**Study Selection:**

Peer-reviewed randomized clinical trials measuring loneliness and social isolation or support in adults aged 65 years or older. Only English language articles were included.

**Data Extraction and Synthesis:**

Two independent reviewers screened studies, extracted data, and assessed risk of bias. Random-effects models were performed to pool the overall effect size by intervention. Statistical heterogeneity was evaluated with the *I*^2^ statistic and by estimating prediction intervals. Data were analyzed from November 2021 to September 2022.

**Main Outcomes and Measures:**

Quantitative measures of loneliness, social isolation, or social support based on an effect size of standardized mean differences.

**Results:**

Seventy studies were included in the systematic review (8259 participants); 44 studies were included in the loneliness meta-analysis (33 in the community with 3535 participants; 11 in long-term care with 1057 participants), with participants’ ages ranging from 55 to 100 years. Study sizes ranged from 8 to 741 participants. Interventions included animal therapy, psychotherapy or cognitive behavioral therapy, multicomponent, counseling, exercise, music therapy, occupational therapy, reminiscence therapy, social interventions, and technological interventions. Most interventions had a small effect size. Animal therapy in long-term care, when accounting for studies with no active controls, had the largest effect size on loneliness reduction (−1.86; 95% CI, −3.14 to −0.59; *I^2^* = 86%) followed by technological interventions (videoconferencing) in long-term care (−1.40; 95% CI, −2.37 to −0.44; *I^2^* = 70%).

**Conclusions and Relevance:**

In this study, animal therapy and technology in long-term care had large effect sizes, but also high heterogeneity, so the effect size’s magnitude should be interpreted with caution. The small number of studies per intervention limits conclusions on sources of heterogeneity. Overall quality of evidence was very low. Future studies should consider measures of social isolation in long-term care and identify the contextual components that are associated with a reduction in loneliness.

## Key Points

**Question:**

What interventions are associated with reduced loneliness and social isolation in older adults?

**Findings:**

In this systematic review of 70 studies with 8259 participants (with meta-analysis of 44 studies with loneliness outcomes; 33 in the community and 11 in long-term care), animal therapy, multicomponent interventions, exercise, technological interventions, and therapy (eg, cognitive behavioral therapy and psychotherapy) had small to large effect sizes associated with reductions in loneliness and social isolation. Studies in long-term care demonstrated a large effect size.

**Meaning:**

These findings suggest that several interventions are associated with a reduction in loneliness in older adults, but cautious interpretation is required given the high heterogeneity and a small number of studies per intervention.

## Teaser

This systematic review and meta-analysis evaluates interventions associated with reduced loneliness and social isolation in older adults.

## Introduction

Older adults (generally defined as those aged ≥65 years)^1,2,3^ are more vulnerable than younger adults to loneliness and social isolation.^4,5,6,7^ The COVID-19 pandemic has exacerbated this phenomenon.^8^ Loneliness is described as the subjective perception of missing social contacts or a desired companion, while social isolation is the objective lack of social contact with other persons.^9^ Loneliness and social isolation are associated with morbidity and mortality.^10,11,12,13^ In the US, one-third of adults aged 45 years and older report loneliness and nearly one-quarter of adults aged 65 years and older are considered socially isolated.^9^

Loneliness in older adults can be mediated by supportive social networks,^14^ physical mobility,^15^ and living arrangements.^9^ Previous systematic reviews (SRs) on interventions targeting loneliness showed that a multitude of interventions^16,17,18,19^ can be associated with reduced loneliness in older adults, including physical exercise,^20,21,22^ reminiscence therapy,^23^ and technological interventions.^24,25^ Several reviews^17,20,21^ incorporated social support as an outcome, suggesting that multiple mechanisms may improve the social milieu of older adults. Although more recent studies have divided interventions by subtype, previous SRs were limited to a specific intervention (eg, exercise or technology),^17,20,21,22,23,24,25^ the absence of meta-analyses, and an older search date.^16,18,26,27^ The most recent meta-analysis^18^ searched the literature to 2009 and included 20 randomized clinical trials of only loneliness outcomes. There has since been increasing awareness of loneliness and social isolation by clinicians, researchers, and policy makers, with calls to centralize evidence and best practices. The National Academies Press additionally highlights the importance of assessing both social isolation and loneliness.^9^ The aim of this SR and meta-analysis was to update and broaden the knowledge base on the interventions associated with a reduction in loneliness and social isolation in older adults.

## Methods

This SR and meta-analysis was registered with PROSPERO (CRD42020178836). We reported according to the Preferred Reporting Items for Systematic Reviews and Meta-analyses (PRISMA) reporting guideline.^39^

### Eligibility Criteria

We included randomized clinical trials of adults aged 65 years and older that reported a validated quantitative outcome measurement of loneliness, social isolation, or social support or network in the English language. There was no exclusion based on prerequisite loneliness and/or social isolation. Theses and protocols were searched to identify subsequently published studies and were included if there was a peer-reviewed journal publication. Social support was defined as a multifaceted concept encompassing the type of support being received, and the perception of having accessible and quality social ties, with social needs being met.^28,29^ Low social support and loneliness are interrelated constructs that are associated with poorer quality of life.^30,31^

### Search Strategy and Selection Criteria

The search was conducted on March 2020. We searched OVID, CINAHL, CENTRAL, Embase, PsychINFO, Web of Science, and Scopus databases. Citations of included SRs were hand searched. We included 5 concepts and their associated MeSH, EMTREE, or PsychINFO terms: older adult, social isolation or loneliness or social support, social intervention, technology, and music therapy or animal therapy. Details of the search strategy can be found in eTable 1 in the Supplement.

### Study Selection

Titles, abstracts, and full-text articles were reviewed in duplicate for inclusion or exclusion. Full texts of intervention studies targeting loneliness or social isolation in older adults were included. Discrepancies were resolved by discussion, and if required, a third reviewer. The κ statistic was used to determine reviewer agreement (eTable 2 in the Supplement) for abstract selection.

### Data Extraction

Data were extracted and entered into an Excel version 16.64 (Microsoft Corp) template by independent pairs of authors (J.M. and S.M., C.V.T. and P.H., K.R. and H.S., C.W. and P.H., and K.M. and P.H.). One author extracted and entered the data while the second author confirmed accuracy. The following data were extracted: author, year, country, setting, study design, number of participants, attrition, demographics (mean or median age and percentage female), inclusion and exclusion criteria, loneliness or social support scale used, study outcomes, and a description of the study groups. Authors were contacted to obtain missing study data. Long-term care (LTC) was defined as participants who required institutional living (eg, nursing home); this excluded assisted and retirement living.^1^ For studies with multiple outcome measurement time points, the final measurement was extracted. Studies were grouped by intervention (eg, animal therapy, psychotherapy or cognitive behavioral therapy [CBT], exercise, social interventions, and information and communications technology), similar to recent SRs.^27,32^ Combination or multicomponent interventions were defined as studies containing multiple different interventions (eg, exercise and CBT).^18,20,24,27^ Intervention types were assessed independently by 1 author (P.H.) and reviewed by 2 other authors (J.M. and J.A.K.). Discrepancies were resolved by consensus.

### Risk of Bias

Risk of bias was assessed independently by pairs of authors (J.M. and S.M., C.V.T. and P.H., K.R. and H.S., C.W. and P.H., and K.M. and P.H.) using the revised Cochrane risk of bias tool for randomized trials.^33^ Discrepancies were resolved by consensus or a third reviewer.

### Statistical Analysis

Articles with quantitative outcomes were included in meta-analysis when possible. Social support and social isolation were analyzed separately from loneliness. The standardized mean difference, Cohen *d*,^34^ and associated 95% CIs were estimated for studies with available data. We used the compute.es package in R version 1.3.1056 (R Project for Statistical Computing) to estimate Cohen *d* when sufficient information was available.^35^ Random-effects models using generic inverse variance methods were performed to pool the overall effect size (ES) by intervention. Statistical heterogeneity was evaluated with the *I*^2^ statistic and estimating prediction intervals. Prediction intervals were estimated using an equation in Higgins et al^36^ that uses a *t* distribution with *K *−* *2 degrees of freedom (where *K* represents number of studies). When heterogeneity was observed, we used the find.outliers function in R to identify which studies may be contributing the most influence to the heterogeneity, then removed these for a subsequent analysis.^37^ Sensitivity analyses were performed excluding multicomponent interventions (eg, combined Tai Chi and CBT) from the main analysis, and studies without active controls. We separately analyzed community and LTC settings, as studies suggest benefit for interventions targeting loneliness in LTC, where loneliness is highly prevalent.^23,32^ Mixed settings that included LTC were not included in the meta-analysis. Heterogeneity was qualitatively assessed given the heterogeneity in study design and methods, and the limited statistical power to perform meta-regression. Funnel plots were produced to assess for potential publication bias (eFigure 1 in the Supplement). Statistical analyses were completed using RStudio, version 1.3.1056 (R Project for Statistical Computing). Estimating methods for Cohen *d* can be found in eTable 3 in the Supplement. The BMJ Best Practice Grading of Recommendations Assessment, Development and Evaluation of Evidence Tool was applied to assess the quality of the evidence.^38^ Two-sided *P* < .05 was considered significant. Data were analyzed from November 2021 to September 2022.

## Results

**Figure 1.**
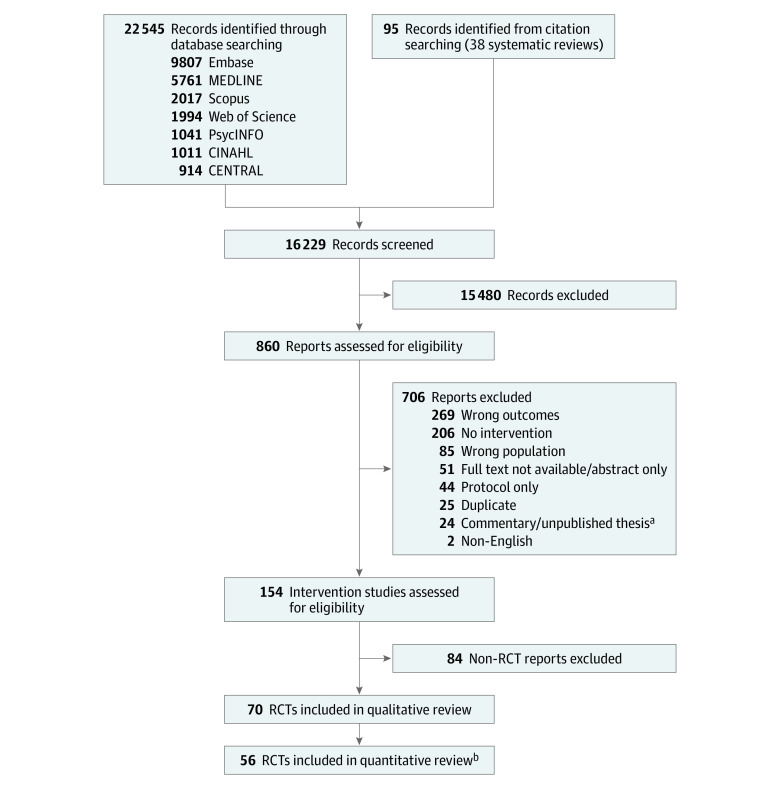
Flow Diagram of Studies Included in the Systematic Review and Meta-analysis RCT indicates randomized clinical trial. ^a^Theses that were not published or do not have an associated publication in a peer-reviewed journal were excluded. ^b^A total of 56 studies were included in quantitative review, comprising 44 studies of loneliness outcomes, 5 studies of social isolation outcomes, and 8 studies of social support outcomes.

The search resulted in 16 229 citations, with 15 460 excluded after title and abstract screening. Eight hundred sixty studies were included for full-text review, of which 790 were excluded. The PRISMA flow diagram of the search results is shown in Figure 1. Seventy studies with 8259 participants met the criteria for inclusion in the SR (Table and eTable 4 in the Supplement). Articles were published between 1985 and 2020. Most studies were conducted in the US (25 studies). Forty-three studies enrolled community-dwelling individuals, and 12 were conducted in LTC settings. Study sizes ranged from 8 to 741 in the SR, with participants being predominantly female (range, 0%-100%) and between the ages of 55 to 100 years. Loneliness was measured using the UCLA Loneliness scale (33 studies),^40^ followed by the De Jong Gierveld Loneliness Scale (13 studies).^41^ Social isolation was primarily measured using the Lubben Social Network Scale (3 studies).^42^

**Table.** Participants and Study Characteristics in Studies Included in the Systematic Review

Source (country)	Setting		Study design	Age, mean (SD), y	Sample, No. (percentage female)			Loneliness/social support scale^**a**^
Animal therapy								
Banks et al,^72^ 2008 (US)	LTC		RCT	NR (NR)	38 (NR)			UCLA
Banks, and Banks,^71^ 2005 (US)	LTC		RCT	80 (NR)	37 (57)			UCLA
Banks and Banks,^70^ 2002 (US)	LTC		RCT	NR (NR)	45 (80)			UCLA
Jessen et al,^75^ 1996 (US)	Rehabilitation unit		RCT	76 (NR)	40 (67.5)			Revised UCLA
Robinson et al,^74^ 2013 (New Zealand)	Retirement home and hospital		RCT	NR (55-100)^b^	40 (67.5)			UCLA
Sollami et al,^73^ 2017 (Italy)	Nursing home		RCT	Intervention: 85.07 (10.12); control: 84.91 (9.07)	28 (NR)			UCLA
Therapy								
Cox et al,^48^ 2007 (US)	Variable		RCT: 3 groups	78.42 (9.78)	177 (76.8)			PGCMS Lonely Dissatisfaction
Jarvis et al,^110^ 2019 (South Africa)	Residential care facility		RCT	74.93 (6.41)	32 (81.3)			De Jong Gierveld (6 item)
Li et al,^63^ 2018 (China)	Community		RCT: Cluster	Intervention: 71.77 (5.49) Control: 71.88 (5.38)	201 (intervention: 44.3; control: 38.6)			Social Support Rating Scale
Nelson et al,^112^ 2019 (US)	Community cancer center		RCT	76 (4)	59 (53)			UCLA Loneliness short form
Parry et al,^65^ 2016 (United Kingdom)	Community		RCT	75.5 (8.55)	415 (70.1)			LSNS (6); De Jong Gierveld
Theeke et al,^111^ 2016 (US)	Community		RCT	75 (7.5)	27 (89)			Revised UCLA
Combination or multicomponent								
Boen et al,^57^ 2012 (Norway)	Community center		RCT	NR (NR)	138 (intervention: 59.5; control: 54.7)			Oslo-3 Social Support Scale
Huang et al,^59^ 2011 (Taiwan)	Community		RCT	NR (NR)	186 (58.6)			Chinese version of the Inventory of Social Supportive Behaviors
Joubert et al,^49^ 2013 (Australia)	Emergency department/community		RCT	71.25 (NR)	8 (75)			MOS: social support
Kapan et al,^58^ 2017 (Austria)	Community		RCT	82.6 (8.1)	80 (84)			WHOQOL-BREF (social support)
Markle-Reid et al,^64^ 2006 (Canada)	Nursing home		RCT	83.82 (5.37)	288 (76.9)			Personal Resource Questionnaire 85 (part 2)
Ollongvist et al,^52^ 2008 (Finland)	Rehabilitation center		RCT	78 (NR)	741 (86)			Subjective 1-4 loneliness scale converted into binary outcome
Saito et al,^77^ 2012 (Japan)	Community		RCT	Intervention: 72.6 (4.4) Control: 72.8 (4.8)	76 (intervention: 60; control: 70)			Ando-Osada-Kodama loneliness scale
Tse et al,^78^ 2012 (Hong Kong)	Nursing home		Cluster RCT	85.17 (6.48)	535 (72.5)			Revised UCLA
Tse et al,^79^ 2013 (China)	Nursing home		RCT: Cluster	≥ 80-89 (NR)^c^	90 (62.2)			Revised UCLA
Tse et al,^80^ 2016 (China)	Nursing home		RCT: Cluster	NR (NR)	60 (82)			Geriatric Suicide Ideation Scale, Chinese version
Counseling								
Alaviani et al,^86^ 2015 (Iran)	Community		RCT	NR (NR)	150 (100)			Revised UCLA
Chow et al,^81^ 2019 (China)	Community		RCT: Cluster	74.3 (7.5)	125 (81.60)			De Jong Gierveld Loneliness Scale (7)
Cohen-Mansfield et al,^82^ 2018 (Israel)	Community		RCT	Intervention: 76.6 (6.8)	89 (81.08)			Mean of 3 weight means: UCLA (8), frequency of loneliness (Mullins), and severity of loneliness
				Control: 79 (6.62)				
Estebsari et al,^43^ 2018 (Iran)	Health house		RCT	65.9 (3.6)	464 (50)			De Jong Gierveld (7)
Kremers et al,^84^ 2006 the (Netherlands)	Community		RCT	Intervention: 62.8. (6.4)	142 (100)			De Jong Gierveld
				Control: 65.2 (7.6)				
Mountain et al,^83^ 2017 (United Kingdom)	Community		RCT	Intervention: 72.9 (65-92)	288 (intervention: 69.7; control: 66.4)			De Jong Gierveld
				Control: 71.3 (65-90)				
Routasalo et al,^85^ 2009 (Finland)	Community		RCT	Intervention: 80 (75-92)^b^^,^^c^	235 (intervention: 74.4; control: 72.9)			UCLA
				Control: 80 (75-90)^b^^,^^c^				
Exercise								
Baez et al,^88^ 2017 (Italy)	Independent living		RCT	71 (5.7)	40 (72.5)			Revised UCLA (3 item)
Chan et al,^90^ 2017 (China)	Community		RCT	77.3 (7.4)	48 (76)			De Jong Gierveld (6)
Ehlers et al,^87^ 2017 (United States)	Community		RCT: 4 groups	65.39 (4.56)	247 (68.4)			UCLA
Jansons et al,^67^ 2017 (Australia)	Community		RCT	Intervention: 68 (11)	105 (63.81)			Friendship Scale
				Control: 66 (13)				
Jones et al,^89^ 2019 (Canada)	Community		RCT	74.5 (6.2)	66 (42.4)			De Jong Gierveld
McAuley, et al^50^ 2000 (US)	Community		RCT	66.71 (5.35)	174 (71.8)			UCLA
Tse et al,^55^ 2014 (China)	Long-term care		RCT	85.44 (6.29)	396 (80.1)			UCLA
Wang et al,^91^ 2010 (US)	Community		RCT	74.9 (8.4)	18 (88.9)			UCLA (3 item)
Music therapy								
Giovagnoli et al,^66^ 2018 (Italy and US)			RCT	73.2 (NR)	45 (68.89)			LSNS
Johnson et al,^92^ 2020 (US)	Senior centers		RCT: Waitlist-control	71.3 (7.2)	390 (76)			National Institutes of Health Toolbox: loneliness
Yap et al,^69^ 2017 (Singapore)	Community		RCT: Waitlist control	74.65 (6.4)	51 (94)			LSNS
Other or miscellaneous								
De Craen et al,^93^ 2006 the (Netherlands)	Community		RCT	85 (NR)	402 (intervention: 64; control: 67)			De Jong Giervield
Larsson et al,^94^ 2016 (Sweden)	Community		RCT: 2-period crossover design	71.2 (NR)	30 (80)			UCLA
Pynnönen et al,^53^ 2018 (Finland)	Community		RCT	77.0 (1.43)	257 (75)			Social provisions scale
Taube et al,^54^ 2018 (Sweden)	Community		RCT	81.5 (6.4)	153 (67)			Single item question
Reminiscence therapy								
Chiang et al,^95^ 2009 (Taiwan)	Nursing home		RCT: Waiting list control	77.24 (3.97)	130 (0)			Revised UCLA
Moieni et al,^97^ 2020 (US)	Community		RCT	70.9 (6.5)	78 (100)			UCLA
Westerhof et al,^96^ 2017 (the Netherlands)	Care facility		RCT	84.2 (8.5)	81 (82)			De Jong Gierveld
Social intervention								
Andersson et al,^100^ 1985 (Sweden)	Community		RCT	77 (NR)	64 (100)			UCLA (4 item)
Charlesworth et al,^102^ 2008 (United Kingdom)	Community		RCT	68 (11.4)	236 (64)			Stroebe 2 item scale; the Multidimensional Scale of Perceived Social Support
Hartke and King.,^101^ 2003 (US)	Community		RCT	69.72 (6)	124 (76)			UCLA
Heller et al,^44^ 1991 (US)	Community		RCT	74 (NR)^c^	291 (100)			Paloutzian/Ellison Loneliness scale
								Perceived social support scale
MacIntyre et al,^61^ 1999 (Canada)	Community		RCT	79.4 (7.0)	22 (68)			Social integration scale
Mountain et al,^99^ 2014 (United Kingdom)	Community		RCT	Intervention: 81.8 (5.8)	70 (58.57)			De Jong Gierveld
				Control: 80.1 (3.7)				
Rook et al,^98^ 2003 (US)	Community		RCT	70.52 (6.89)	180 (65.6)			UCLA (10 item)
Walshe et al,^56^ 2016 (United Kingdom)	Community		RCT	72 (37-92)^b^	196 (60)			De Jong Gierveld (6)
Technology								
Bickmore et al,^103^ 2005 (US)	Community		RCT	74 (NR)	21 (86)			Revised UCLA
Bond et al,^62^ 2010 (US)	Community		RCT	Intervention: 66 (5.7)	62 (45)			Diabetes support scale
				Control: 68 (6.2)				
Czaja et al,^104^ 2018 (US)	Community		RCT	76.15 (7.4)	300 (78)			UCLA-V3
Dodge et al,^105^ 2015 (US)	Retirement community and senior center		RCT	80.5 (6.8)	83 (75.9)			Hughs loneliness scale
Gustafson et al,^76^ 2019 (US)			RCT	NR (NR)	31 (61.3)			UCLA Loneliness Scale
Morgenstern et al,^68^ 2015 (US)	Community		RCT	Intervention: 76.95 (8.51)	265 (100)			Perceived Isolation Index in an elderly population
				Control: 75.05 (8.20)				
Morton et al,^46^ 2018 (Australia)	Community and Care homes		RCT: 2x2	80.71 (8.77)	121 (65)			UCLA (8 item)
Nikitina et al,^51^ 2018 (Russia)	Community		RCT	Pilot 1: Intervention 68.2 (7.8)	Pilot 1: 20 (95)			Revised UCLA (3 item)
				Control: 65.0 (6.1)	Pilot 2: 40 (100)			
				Pilot 2: Intervention 67.6 (6.2)				
				Control: 68.8 (7.2)				
Sidner et al,^106^ 2018 (US)	Community		RCT: 3 groups	66 (7.89)	44 (NR)			Revised UCLA
Slegers et al,^107^ 2008 (the Netherlands)	Community		RCT: multigroup control	NR (NR)	236 (NR)			De Jong Gierveld
Tsai et al,^108^ 2011 (Taiwan)	Nursing home		RCT	Intervention: 73.82 (11.19)	90 (intervention: 55; control: 60)			UCLA Loneliness Scale
				Control: 79.26 (7.07)				
Tsai et al,^109^ 2020 (China)	Long-term care		RCT: cluster	Intervention: 81.07 (8.46)	62 (intervention: 75; control: 56.7)			Revised UCLA
				Control: 68.95 (11.65)				
Wan et al,^60^ 2017 (US)	Community		RCT	68.6 (8.3)	114 (1.8)			MOS: Social Support
White et al,^47^ 2002 (US)	Congregate housing and nursing facility		RCT	Intervention: 71 (12)	100 (intervention: 71; control: 82)			UCLA
				Control: 72 (11)				
Woodward et al,^45^ 2011 (US)	Community		RCT	71.85 (7.09)	83 (72)			Loneliness was measured using a 6 item scale

Abbreviations: De Jong Gierveld, De Jong Gierveld Loneliness Scale; LSNS, Lubben Social Network Scale; LTC, long-term care; MOS, Medical outcomes study social support survey; NR, not reported; PGCMS, Philadelphia Geriatric Center Morale Scale; RCT, randomized clinical trial; UCLA, University of California, Los Angeles Loneliness Scale.

^a^ Short versions of the scale are identified by the number of items in parenthesis next to the scale name.

^b^ Denotes a range.

^c^ Denotes a median.

**Figure 2.**
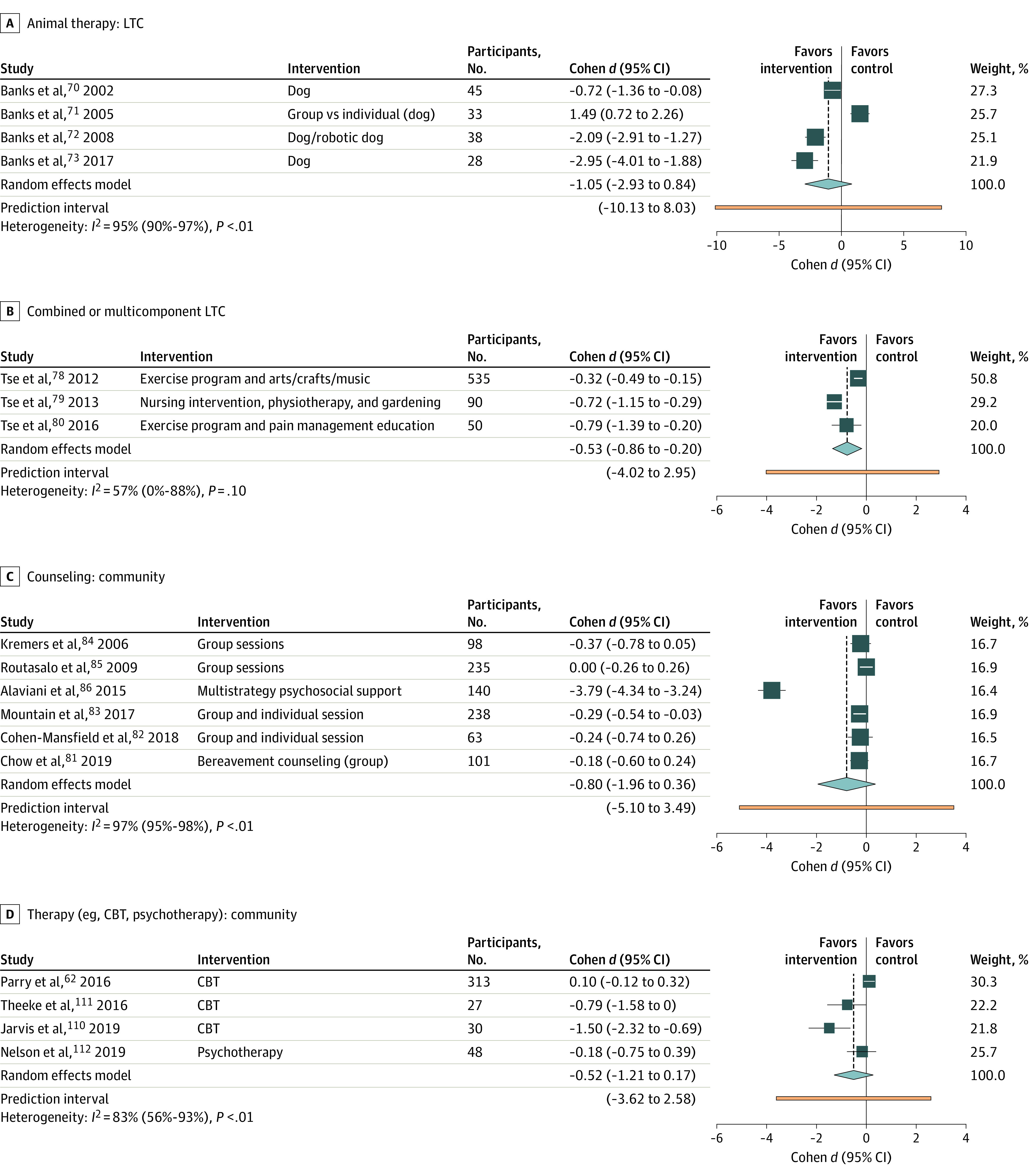
Meta-analysis Forest Plot Summary Divided by Intervention: Animal Therapy, Combined or Multicomponent, Counseling, and Cognitive Behavioral Therapy and Psychotherapy CBT indicates cognitive behavioral therapy; LTC, long-term care.

**Figure 3.**
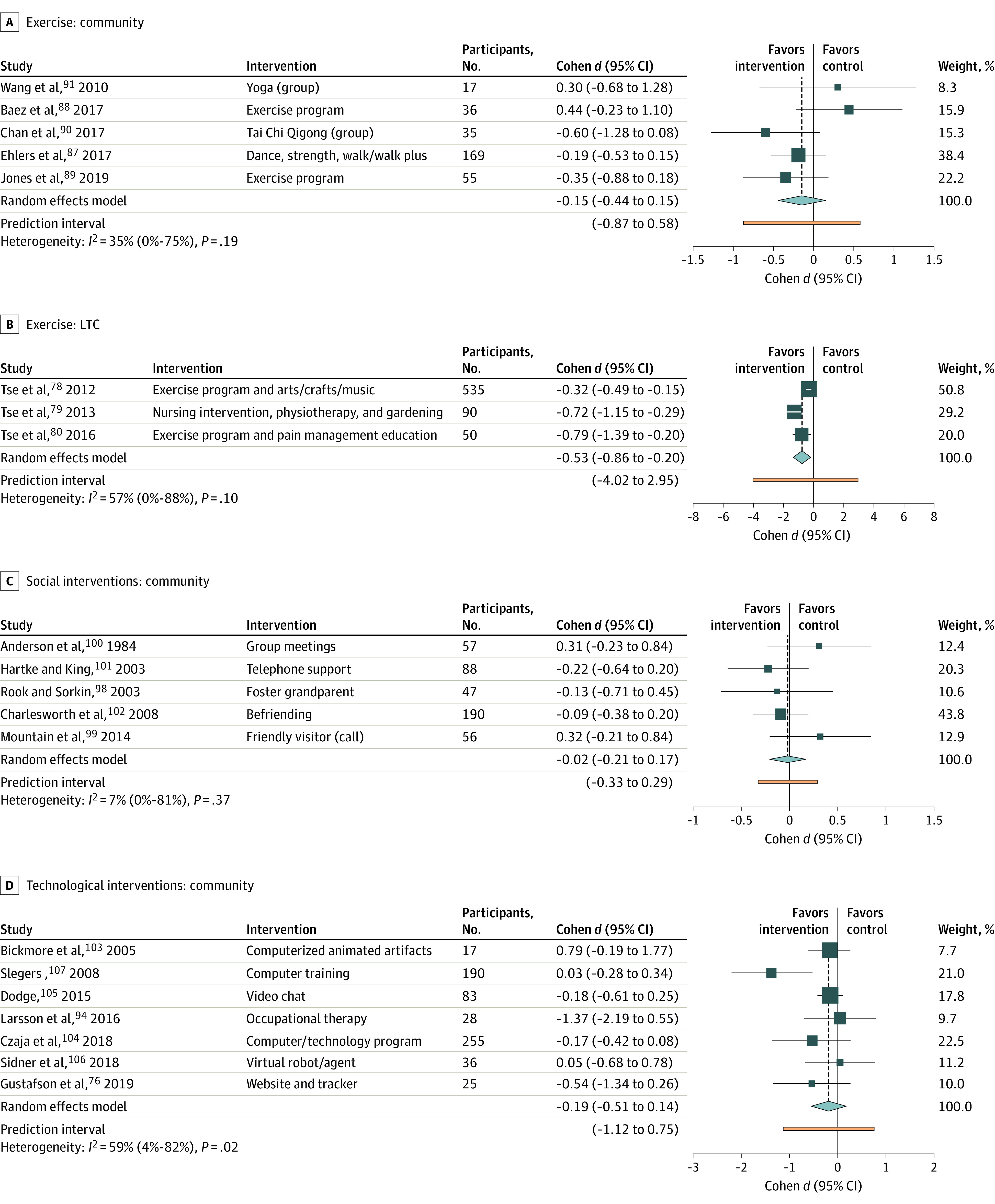
Meta-analysis Forest Plot Summary Divided by Intervention: Exercise, Social Interventions, and Technological Interventions LTC indicates long-term care.

**Figure 4.**
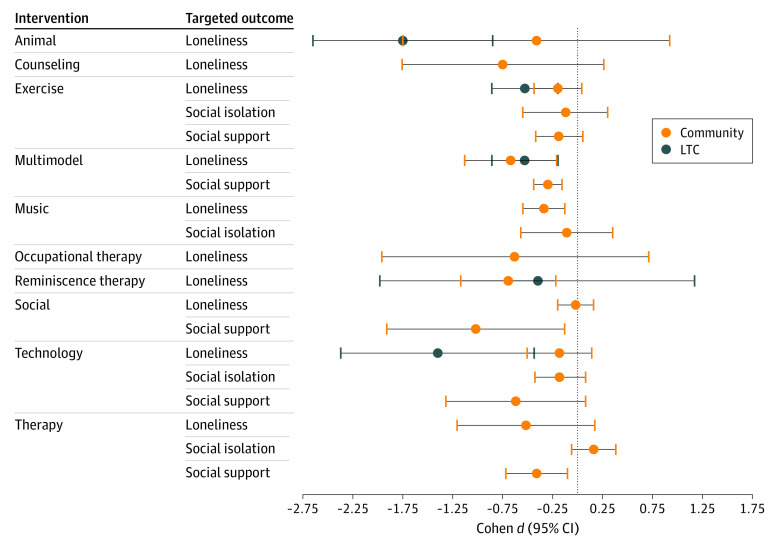
Summary of Meta-analysis Data Including Loneliness, Social Isolation, and Social Support, Stratified by Setting (Community and Long-Term Care [LTC]) Social support outcome has been inverted such that benefit is toward the left of the figure.

Fourteen studies were excluded from the meta-analysis for lack of reported outcomes or LTC combined with a community setting (eTable 5 in the Supplement).^43,44,45,46,47,48,49,50,51,52,53,54,55,56^ Forty-four studies were included in the loneliness outcome meta-analysis (33 in community; 11 in LTC) (Figure 2, Figure 3, and Figure 4; eFigure 2 in the Supplement). The social support outcome meta-analysis (8 studies), all set in the community, is found in eFigure 2 and eTable 6 in the Supplement.^57,58,59,60,61,62,63,64^ Five studies set in the community measured social isolation (eFigure 2, eTable 6 in the Supplement).^65,66,67,68,69^ The total number of participants eligible for loneliness quantitative analysis was 3535 in community and 1057 in LTC (706 participants for social isolation [community] and 932 participants for social support [community]). Meta-analyses are presented for loneliness outcomes unless otherwise specified. Forest plots with 2 or fewer studies can be found in eFigure 2 in the Supplement. Seven studies had a loneliness enrollment prerequisite. Overall study quality was very low (eTable 7 in the Supplement). The overall risk of bias of the included studies was high (eFigure 3 and eTable 8 in the Supplement), associated with the effect of adhering to the intervention (eg, participants’ and researchers’ awareness of the intervention), unreported adherence outcomes, and repeated outcome measurement.

### Animal Therapy

Six studies^70,71,72,73,74,75^ were included in the meta-analysis, 2 in the community (Figure 4) and 4 in LTC with an ES of −0.41 (95% CI, −1.75 to 0.92; *I^2^* = 87%; *P* = .005) and −1.05 (95% CI, −2.93 to 0.84; *I^2^* = 95%; *P* < .001), respectively. Upon excluding a study^71^ comparing group to individual animal therapy, the effect size was −1.86 (95% CI, −3.14 to −0.59; *I^2^* = 86%; *P* < .001). Generally, participants interacted with living dogs or robotic animals (seal or dog). One study^75^ provided a bird in the participant’s room for the study duration.

### Combination and Multicomponent Interventions

Five studies^76,77,78,79,80^ were included in the meta-analysis, 2 in the community (Figure 4) and 3 in LTC. The ES was −0.67 (95% CI, −1.13 to −0.21; *I^2^* = 0%; *P* = .704) in community and −0.53 (95% CI, −0.86 to −0.20; *I^2^* = 57%; *P* = .099) in LTC. Interventions included exercise with arts and crafts, home care with nursing outreach and educational resources, Tai Chi and CBT, and pain management programs. Six studies^57,58,59,60,63,64^ were included in social support meta-analysis (all community-dwelling), with an ES of 0.29 (95% CI, 0.15 to 0.43) and low heterogeneity (*I^2^* = 0%; *P* = .66).

### Counseling

Six group-based studies^81,82,83,84,85,86^ in community-dwelling participants were included in the meta-analysis. Interventions included bereavement counseling and instructor-led group support programs. The ES was −0.80 (95% CI, −1.96 to 0.36); heterogeneity was substantial (*I^2^* = 97%; *P* < .001). When excluding Alaviani et al,^86^ the ES was less pronounced (−0.19; 95% CI, −0.35 to −0.03), with no heterogeneity (*I^2^* = 0%; *P* = .48).

### Exercise

Eight studies^78,79,80,87,88,89,90,91^ were included in the meta-analysis, 5 in the community and 3 in LTC. All but 1 study was performed in a group setting (dance, yoga, Tai Chi, and strength and balance training).^67^ The ES was −0.15 (95% CI, −0.44 to 0.15) and heterogeneity was low (*I^2^* = 35%; *P* = .19) in community and −0.53 (95% CI, −0.86 to −0.20; *I^2^* = 57%; *P* = .10) in LTC. The ES was similar when excluding studies without active controls (−0.45 in community; −0.32 in LTC). Three studies^57,58,59^ were included in social support meta-analysis; ES was 0.17 (95% CI, −0.07 to 0.41) with low heterogeneity (*I^2^* = 0%; *P* = .48). There was a potential for small study publication bias on the community funnel plot (eFigure 1 in the Supplement). One study^67^ set in the community assessed social isolation, with an ES of −0.12 (95% CI, −0.55 to 0.31).

### Music

One community study^92^ measured loneliness with an ES of −0.34 (95% CI, −0.55 to −0.13). Two community studies^66,69^ were included in the meta-analysis for social isolation. The ES was −0.11 (95% CI, −0.57 to 0.35) and heterogeneity was low (*I^2^* = 0.0%; *P* = .37). Interventions included group rhythm instruments and a choir program.

### Occupational Therapist–Guided Interventions

Two community-based studies^93,94^ were included in the meta-analysis (Figure 4). Interventions included occupational therapist-guided technology or assistive devices training. The ES was −0.63 (95% CI, −1.96 to 0.71) with substantial heterogeneity (*I^2^* = 90%; *P* = .002). Larsson et al,^94^ which enrolled persons who were lonely at baseline, was the only study that had a significant outcome in reducing loneliness.

### Reminiscence Therapy

Two studies^95,96^ set in LTC were included in the meta-analysis (Figure 4). Interventions included volunteer-led individual reminiscence sessions to group sessions that included sharing memories and identifying goals. The ES was −0.40 (95% CI, −1.98 to 1.17); heterogeneity was substantial (*I^2^* = 95%; *P* < .001). The study by Moieni et al,^97^ which was set in the community, showed similar findings (ES −0.70; 95% CI, −1.17 to −0.22).

### Social Intervention

Five studies^98,99,100,101,102^ set in the community were included in the meta-analysis. Interventions included befriending a volunteer, formation of social groups with discussion topics, and intergenerational programming. The ES was −0.02 (95% CI, −0.21 to 0.17) with low heterogeneity (*I*^2^ = 7%; *P* = .37). Excluding studies without active controls (ES = 0.10; 95% CI, −0.27 to 0.48) did not alter the conclusions. One study set in the community showed significant improvement in social support (ES = 1.02; 95% CI, 0.13 to 1.91).^61^

### Technology

Nine studies^76,94,103,104,105,106,107,108,109^ were included in the meta-analysis, 7 in the community and 2 in LTC (Figure 4). Interventions included computer training (in-person or online), videoconferencing (either with family or a trained interviewer), and pedometers to track and provide fitness goals. The ES was −0.19 (95% CI, −0.51 to 0.14; *I^2^* = 59%; *P* = .03) in community and −1.40 (95% CI, −2.37 to −0.44; *I^2^* = 70%; *P* = .07) in LTC. Attrition was highest in studies in LTC (up to 44%).^108,109^ Exclusion of multicomponent interventions (community) did not change the ES (−0.15; 95% CI, −0.53 to 0.24). Social support meta-analysis (2 studies), set in the community-dwelling, had an ES of 0.62 (95% CI, −0.07 to 1.31; *I^2^* = 78%; *P* = .03).^60,62^ One community-based study^68^ measured social isolation (ES, −0.18; 95% CI, −0.43 to 0.08).

### CBT and Psychotherapy

Four studies^65,110,111,112^ set in the community were included in the meta-analysis with an ES of −0.52 (95% CI, −1.21 to 0.17), provided by trained personnel (eg, psychotherapist, doctoral students) in individual and group sessions. There was considerable heterogeneity (*I^2^* = 83%; *P* < .001). Upon excluding studies without active controls, the ES remained similar at −0.46 (95% CI, −1.39 to 0.46). One study^63^ measured social support in the community (ES, 0.41; 95% CI, 0.10 to 0.72). Parry et al^65^ also measured social isolation, with an ES of 0.16 (95% CI, −0.06 to 0.38).

## Discussion

This SR and meta-analysis aimed to determine which interventions are associated with a reduction in loneliness and social isolation in older adults and, to our knowledge, is the largest and most comprehensive such study to date. We additionally separately analyzed outcomes of loneliness, social isolation, and social support. Overall, we found that animal therapy (accounting for one study of group compared to individual animal therapy) and technological interventions in LTC had a large ES. However, given the small number of studies in each intervention and high heterogeneity, cautious interpretation of the ES’s magnitude is required.

Many interventions are associated with a reduction in loneliness, and all encouraged socialization in some form. Expectations and perceptions of an intervention may influence its effectiveness.^113^ For example, studies with a specific goal, particularly in a group setting, may build social relationships that are associated with less loneliness (eg, exercise, multicomponent interventions, animal therapy, technology, psychotherapy, and CBT).^27^ However, group interventions are not necessarily beneficial.^27,114^ Interventions that target coping strategies (eg, psychotherapy, counseling, CBT, or reminiscence) may modify individual and environmental factors that can influence social behavior and self-efficacy, thereby reducing loneliness and improving socialization.^115,116,117^

Perhaps surprisingly, social interventions were not significant. Although social prescribing is thought to be a potentially effective intervention, in some it may cause social anxiety.^118,119,120^ Additionally, meaningful friendships may not always result from prescribed interventions. Studies of longer duration, required to develop strong friendships, or high value relationships (eg, family and close friends) should be considered and prioritized.^121^ Although this may explain why online interactions (eg, videoconferencing) with family had a large ES, only 2 studies were included. Accordingly, it is important that socially prescribed interventions are tailored to an individual’s unique needs. Interventions in LTC may have shown greater ESs for several reasons, including a higher prevalence of loneliness, and being accustomed to group living with shared programs and activities.^32,122,123,124^ A number of interventions among community-dwelling participants were not associated with reduced loneliness, potentially related to a highly heterogeneous population.^125,126^ Interventions with social support meta-analysis generally had a smaller ES. This may reflect the subjective nature of these experiences and that an intervention may not change an individual’s social network despite reducing their loneliness.^29,31^

Our SR found similar findings to previous studies. Animal therapy has generally shown positive outcomes for loneliness, potentially mediated by previous pet ownership (particularly dogs).^17,127,128^ Multicomponent interventions generally found success, which is not unexpected given the advantage of incorporating multiple interventions.^19^ Due to the multicomponent design of the LTC studies of exercise, the outcomes of exercise could not be isolated, despite the consistent negative effect size. Moreover, only 3 studies were identified. Similarly, reviews of exercise have found conflicting evidence on social support and loneliness.^20,21,22^ Reviews that included reminiscence therapy found benefit, but few studies were identified.^18,19,23^ Reviews of technological interventions generally found mixed ESs on loneliness.^18,19,114,129,130^ Although a review from Chipps et al^24^ identified benefit for videoconferencing, we have shown that this is potentially associated with studies in LTC. We similarly found no benefit for technological training programs (community). Similar to other reviews, there was moderate to substantial heterogeneity across most studies, which may be due to the complex and individual nature of loneliness.^119,131^

Interventions targeting negative self-thought were not consistently associated with reduced loneliness, though evidence suggests such interventions may be beneficial.^132^ Future RCTs should aim for more equitable representation of sex/gender and culture, and incorporate measures of loneliness and social isolation.^133^ Social isolation and social support require further study in LTC. Future studies should consider identifying important contextual components in LTC associated with a reduction in loneliness. Methodological quality can be improved by adherence outcomes and its analyses, reporting adverse events, and implementing active controls.

### Limitations

This study has limitations. Sample sizes were small, and few studies used active controls, the latter potentially confounding the intervention effect. The majority of studies enrolled community-dwelling older adults and may affect generalizability. A number of studies of specific interventions shared study authors and participant recruitment geographical locations, which may increase the risk of bias: all studies of exercise and multicomponent in LTC (Tse et al),^78,79,80^ 3 of 4 studies of animal therapy in LTC (Banks et al),^70,71,72^ and all studies of technology in LTC (Tsai et al).^108,109^ The latter 2 authors reported no overlap in study samples in our correspondence. Although Tse et al reported separately collected cohorts, the possibility of participant overlap remains. The small number of studies per meta-analysis limited conclusions on sources of heterogeneity and the ES’s magnitude. The sustainability of the effect of the interventions cannot be concluded on the basis of our results. Only English language studies were included. Several studies required estimating Cohen *d* for pooling based on data provided in the original study (eg, *P* values and 95% CIs).

## Conclusions

In this SR and meta-analysis, exercise and technological interventions in the community had the highest precision with small ESs, whereas animal therapy in LTC had the largest ES when accounting for one study comparing group to individual therapy. When exercise is combined with other interventions (eg, CBT), the benefit may be strengthened. These results require cautious interpretation due to high heterogeneity and a small number of studies, particularly with respect to the ES’s magnitude.

## Notes

Supplement. **eTable 1. **Search Strategy With Each Corresponding Database **eTable 2. **Cohen κ for Reviewed Abstracts **eTable 3. **Estimating Methods for Cohen *d* (Effect Sizes)
eReferences
**eTable 4. **Intervention and Control Group Characteristics of Included Studies **eTable 5. **Reasons for Exclusion From Meta-analysis **eTable 6. **Meta-analyses by Intervention and Sensitivity Analyses **eTable 7. **GRADE Table of Included Studies **eTable 8. **Risk of Bias Table of Included Studies **eFigure 1. **Funnel Plot Analysis of Studies Included in Meta-analysis **eFigure 2. **Risk of Bias Assessment of Studies Included in the Systematic Review

## References

[1] National Center for Health Statistics. Vital and health statistics. series 3, number 43. 2019. Accessed June 21, 2022. https://www.cdc.gov/nchs/products/index.htm

[2] National Prevention Council. Healthy aging in action: advancing the national prevention strategy. 2016. Accessed June 23, 2022. https://www.cdc.gov/aging/pdf/healthy-aging-in-action508.pdf 30896903

[3] Singh S, Bajorek B. Defining ‘elderly’ in clinical practice guidelines for pharmacotherapy. Pharm Pract (Granada). 2014;12(4):489. doi:10.4321/S1886-36552014000400007 25580172 PMC4282767

[4] Social Care Institute for Excellence. At a glance 60: preventing loneliness and social isolation among older people. 2012. Accessed March 2, 2022. https://www.scie.org.uk/publications/ataglance/ataglance60.asp

[5] Bernard S, Perry H. Loneliness and social isolation among older people in north yorkshire: executive summary. York. 2013. Accessed March 2, 2022. https://eprints.whiterose.ac.uk/77336/1/Lone.pdf

[6] Centers for Disease Control and Prevention. Loneliness and social isolation linked to serious health conditions. Accessed September 28, 2022. https://www.cdc.gov/aging/publications/features/lonely-older-adults.html

[7] Dahlberg L, McKee KJ, Frank A, Naseer M. A systematic review of longitudinal risk factors for loneliness in older adults. Aging Ment Health. 2022;26(2):225-249. doi:10.1080/13607863.2021.1876638 33563024

[8] Wu B. Social isolation and loneliness among older adults in the context of COVID-19: a global challenge. Glob Heal Res Policy. 2020;5(1):1-3. doi:10.1186/s41256-020-00154-3 32514427 PMC7272234

[9] National Academies of Sciences and Medicine Engineering. Social Isolation and Loneliness in Older Adults: Opportunities for the Health Care System. The National Academies Press; 2020. doi:10.17226/25663. 32510896

[10] Hawkley LC, Cacioppo JT. Aging and loneliness: downhill quickly? Curr Dir Psychol Sci. 2007;16(4):187-191. doi:10.1111/j.1467-8721.2007.00501.x

[11] Cacioppo JT, Cacioppo S. Older adults reporting social isolation or loneliness show poorer cognitive function 4 years later. Evid Based Nurs. 2014;17(2):59-60. doi:10.1136/eb-2013-101379 23749730

[12] Lee SL, Pearce E, Ajnakina O, . The association between loneliness and depressive symptoms among adults aged 50 years and older: a 12-year population-based cohort study. Lancet Psychiatry. 2021;8(1):48-57. doi:10.1016/S2215-0366(20)30383-7 33181096 PMC8009277

[13] Golaszewski NM, LaCroix AZ, Godino JG, . Evaluation of social isolation, loneliness, and cardiovascular disease among older women in the US. JAMA Netw Open. 2022;5(2):e2146461-e2146461. doi:10.1001/jamanetworkopen.2021.46461 35107574 PMC8811637

[14] Kim H, Kwak S, Youm Y, Chey J. Social network characteristics predict loneliness in older adults. Gerontology. 2022;68(3):309-320. doi:10.1159/000516226 34182553 PMC8985000

[15] Schrempft S, Jackowska M, Hamer M, Steptoe A. Associations between social isolation, loneliness, and objective physical activity in older men and women. BMC Public Health. 2019;19(1):74. doi:10.1186/s12889-019-6424-y 30651092 PMC6335852

[16] Dickens AP, Richards SH, Greaves CJ, Campbell JL. Interventions targeting social isolation in older people: a systematic review. BMC Public Health. 2011;11:647. doi:10.1186/1471-2458-11-647 21843337 PMC3170621

[17] Gee NR, Mueller MK. A systematic review of research on pet ownership and animal interactions among older adults. Anthrozoös. 2019;32(2):183-207. doi:10.1080/08927936.2019.1569903

[18] Masi CM, Chen HY, Hawkley LC, Cacioppo JT. A meta-analysis of interventions to reduce loneliness. Pers Soc Psychol Rev. 2011;15(3):219-266. doi:10.1177/1088868310377394 20716644 PMC3865701

[19] Poscia A, Stojanovic J, La Milia DI, . Interventions targeting loneliness and social isolation among the older people: an update systematic review. Exp Gerontol. 2018;102:133-144. doi:10.1016/j.exger.2017.11.017 29199121

[20] Snowden MB, Steinman LE, Carlson WL, . Effect of physical activity, social support, and skills training on late-life emotional health: a systematic literature review and implications for public health research. Front Public Health. 2015;2:213. doi:10.3389/fpubh.2014.00213 25964921 PMC4410348

[21] Shvedko A, Whittaker AC, Thompson JL, Greig CA. Physical activity interventions for treatment of social isolation, loneliness or low social support in older adults: a systematic review and meta-analysis of randomised controlled trials. Psychol Sport Exerc. 2018;34:128-137. doi:10.1016/j.psychsport.2017.10.003

[22] Pels F, Kleinert J. Loneliness and physical activity: a systematic review. Int Rev Sport Exerc Psychol. 2016;9(1):231-260. doi:10.1080/1750984X.2016.1177849 PMC4706019 26807143

[23] Syed Elias SM, Neville C, Scott T. The effectiveness of group reminiscence therapy for loneliness, anxiety and depression in older adults in long-term care: a systematic review. Geriatr Nurs. 2015;36(5):372-380. doi:10.1016/j.gerinurse.2015.05.004 26099638

[24] Chipps J, Jarvis MA, Ramlall S. The effectiveness of e-interventions on reducing social isolation in older persons: a systematic review of systematic reviews. J Telemed Telecare. 2017;23(10):817-827. doi:10.1177/1357633X17733773 28958209

[25] Nnabuko U, Anderson S. A systematic review on the effect of ICT on social support measures in healthcare. In: Macedo M, ed. IADIS International Conference on e-Health. 2017:117-128. Accessed September 7, 2022. http://www.iadisportal.org/digital-library/a-systematic-review-on-the-effect-of-ict-on-social-support-measures-in-healthcare

[26] Stojanovic J, Collamati A, Mariusz D, . Decreasing loneliness and social isolation among the older people: systematic search and narrative review. Epidemiol Biostat Public Health. 2017;14(2):1-8. doi:10.2427 /12408

[27] Gardiner C, Geldenhuys G, Gott M. Interventions to reduce social isolation and loneliness among older people: an integrative review. Health Soc Care Community. 2018;26(2):147-157. doi:10.1111/hsc.12367 27413007

[28] Hupcey JE. Clarifying the social support theory-research linkage. J Adv Nurs. 1998;27(6):1231-1241. doi:10.1046/j.1365-2648.1998.01231.x 9663875

[29] Wang J, Mann F, Lloyd-Evans B, Ma R, Johnson S. Associations between loneliness and perceived social support and outcomes of mental health problems: a systematic review. BMC Psychiatry. 2018;18(1):156. doi:10.1186/s12888-018-1736-5 29843662 PMC5975705

[30] Joyce J, Ryan J, Owen A, ; ASPREE Investigator Group. Social isolation, social support, and loneliness and their relationship with cognitive health and dementia. Int J Geriatr Psychiatry. Published online November 5, 2021. doi:10.1002/gps.5644 34741340 PMC9068834

[31] Freak-Poli R, Ryan J, Tran T, . Social isolation, social support and loneliness as independent concepts, and their relationship with health-related quality of life among older women. Aging Ment Heal. 2022;26(7):1335-1344. doi:10.1080/13607863.2021.1940097 34219569

[32] Quan NG, Lohman MC, Resciniti NV, Friedman DB. A systematic review of interventions for loneliness among older adults living in long-term care facilities. Aging Ment Health. 2020;24(12):1945-1955. doi:10.1080/13607863.2019.1673311 31602993

[33] Higgins J, Thomas J, Chandler J, , eds. Cochrane Handbook for Systematic Reviews of Interventions Version 6.3. 2nd ed. John Wiley & Sons; 2019. doi:10.1002/9781119536604

[34] Cohen J. Statistical Power Analysis for the Behavioral Sciences. 2nd ed. Lawrence Erlbaum Associates; 2013.

[35] Del Re A. compute.es: compute effect sizes. 2020. Accessed March 2, 2022. https://cran.r-project.org/web/packages/compute.es/index.html

[36] Higgins JPT, Thompson SG, Spiegelhalter DJ. A re-evaluation of random-effects meta-analysis. J R Stat Soc Ser A Stat Soc. 2009;172(1):137-159. doi:10.1111/j.1467-985X.2008.00552.x 19381330 PMC2667312

[37] Harrer M, Cuijpers P, Furukawa TA, Ebert DD. Doing Meta-Analysis With R: A Hands-On Guide. Chapman & Hall/CRC Press; 2021. Accessed November 22, 2021. https://www.routledge.com/Doing-Meta-Analysis-with-R-A-Hands-On-Guide/Harrer-Cuijpers-Furukawa-Ebert/p/book/9780367610074. doi:10.1201/9781003107347

[38] Guyatt GH, Oxman AD, Vist GE, ; GRADE Working Group. GRADE: an emerging consensus on rating quality of evidence and strength of recommendations. BMJ. 2008;336(7650):924-926. doi:10.1136/bmj.39489.470347.AD 18436948 PMC2335261

[39] Page MJ, McKenzie JE, Bossuyt PM, . The PRISMA 2020 statement: an updated guideline for reporting systematic reviews. BMJ. 2021;372:n71. doi:10.1136/bmj.n71 33782057 PMC8005924

[40] Russell DW. UCLA Loneliness Scale (version 3): reliability, validity, and factor structure. J Pers Assess. 1996;66(1):20-40. doi:10.1207/s15327752jpa6601_2 8576833

[41] de Jong-Gierveld J, Kamphuls F. The development of a Rasch-type loneliness scale. Appl Psychol Meas. 1985;9:289-299. doi:10.1177/014662168500900307

[42] Lubben JE. Assessing social networks among elderly populations. Fam Community Health. 1988;11(3):42-52. doi:10.1097/00003727-198811000-00008

[43] Estebsari F, Dastoorpoor M, Mostafaei D, . Design and implementation of an empowerment model to prevent elder abuse: a randomized controlled trial. Clin Interv Aging. 2018;13:669-679. doi:10.2147/CIA.S158097 29713151 PMC5909776

[44] Heller K, Thompson MG, Trueba PE, Hogg JR, Vlachos-Weber I. Peer support telephone dyads for elderly women: was this the wrong intervention? Am J Community Psychol. 1991;19(1):53-74. doi:10.1007/BF00942253 1867151

[45] Woodward AT, Freddolino PP, Blaschke-Thompson CM, . Technology and aging project: training outcomes and efficacy from a randomized field trial. Ageing Int. 2011;36(1):46-65. doi:10.1007/s12126-010-9074-z

[46] Morton TA, Wilson N, Haslam C, Birney M, Kingston R, McCloskey LG. Activating and guiding the engagement of seniors with online social networking: experimental findings from the AGES 2.0 project. J Aging Health. 2018;30(1):27-51. doi:10.1177/0898264316664440 27530332

[47] White H, McConnell E, Clipp E, . A randomized controlled trial of the psychosocial impact of providing internet training and access to older adults. Aging Ment Health. 2002;6(3):213-221. doi:10.1080/13607860220142422 12217089

[48] Cox EO, Green KE, Hobart K, Jang LJ, Seo H. Strengthening the late-life care process: effects of two forms of a care-receiver efficacy intervention. Gerontologist. 2007;47(3):388-397. doi:10.1093/geront/47.3.388 17565103

[49] Joubert L, Lee J, McKeever U, Holland L. Caring for depressed elderly in the emergency department: establishing links between sub-acute, primary, and community care. Soc Work Health Care. 2013;52(2-3):222-238. doi:10.1080/00981389.2012.737896 23521386

[50] McAuley E, Blissmer B, Marquez DX, Jerome GJ, Kramer AF, Katula J. Social relations, physical activity, and well-being in older adults. Prev Med. 2000;31(5):608-617. doi:10.1006/pmed.2000.0740 11071843

[51] Nikitina S, Didino D, Baez M, Casati F. Feasibility of virtual tablet-based group exercise among older adults in Siberia: findings from two pilot trials. JMIR Mhealth Uhealth. 2018;6(2):e40. doi:10.2196/mhealth.7531 29487045 PMC5849795

[52] Ollonqvist K, Palkeinen H, Aaltonen T, . Alleviating loneliness among frail older people—findings from a randomised controlled trial. Int J Ment Health Promot. 2008;10(2):26-34. doi:10.1080/14623730.2008.9721760

[53] Pynnönen K, Törmäkangas T, Rantanen T, Tiikkainen P, Kallinen M. Effect of a social intervention of choice vs. control on depressive symptoms, melancholy, feeling of loneliness, and perceived togetherness in older Finnish people: a randomized controlled trial. Aging Ment Health. 2018;22(1):77-84. doi:10.1080/13607863.2016.1232367 27657351

[54] Taube E, Kristensson J, Midlöv P, Jakobsson U. The use of case management for community-dwelling older people: the effects on loneliness, symptoms of depression and life satisfaction in a randomised controlled trial. Scand J Caring Sci. 2018;32(2):889-901. doi:10.1111/scs.12520 28895175

[55] Tse MMY, Tang SK, Wan VTC, Vong SKS. The effectiveness of physical exercise training in pain, mobility, and psychological well-being of older persons living in nursing homes. Pain Manag Nurs. 2014;15(4):778-788. doi:10.1016/j.pmn.2013.08.003 24361207

[56] Walshe C, Dodd S, Hill M, . How effective are volunteers at supporting people in their last year of life? a pragmatic randomised wait-list trial in palliative care (ELSA). BMC Med. 2016;14(1):203. doi:10.1186/s12916-016-0746-8 27931214 PMC5146890

[57] Bøen H, Dalgard OS, Johansen R, Nord E. A randomized controlled trial of a senior centre group programme for increasing social support and preventing depression in elderly people living at home in Norway. BMC Geriatr. 2012;12:20. doi:10.1186/1471-2318-12-20 22607553 PMC3494554

[58] Kapan A, Winzer E, Haider S, . Impact of a lay-led home-based intervention programme on quality of life in community-dwelling pre-frail and frail older adults: a randomized controlled trial. BMC Geriatr. 2017;17(1):154. doi:10.1186/s12877-017-0548-7 28724351 PMC5517808

[59] Huang TT, Yang LH, Liu CY. Reducing the fear of falling among community-dwelling elderly adults through cognitive-behavioural strategies and intense Tai Chi exercise: a randomized controlled trial. J Adv Nurs. 2011;67(5):961-971. doi:10.1111/j.1365-2648.2010.05553.x 21214623

[60] Wan ES, Kantorowski A, Homsy D, . Promoting physical activity in COPD: insights from a randomized trial of a web-based intervention and pedometer use. Respir Med. 2017;130:102-110. doi:10.1016/j.rmed.2017.07.057 29206627 PMC5718161

[61] MacIntyre I, Corradetti P, Roberts J, Browne G, Watt S, Lane A. Pilot study of a visitor volunteer programme for community elderly people receiving home health care. Health Soc Care Community. 1999;7(3):225-232. doi:10.1046/j.1365-2524.1999.00178.x 11560637

[62] Bond GE, Burr RL, Wolf FM, Feldt K. The effects of a web-based intervention on psychosocial well-being among adults aged 60 and older with diabetes: a randomized trial. Diabetes Educ. 2010;36(3):446-456. doi:10.1177/0145721710366758 20375351

[63] Li X, Wang B, Tan D, . Effectiveness of comprehensive social support interventions among elderly patients with tuberculosis in communities in China: a community-based trial. J Epidemiol Community Health. 2018;72(5):369-375. doi:10.1136/jech-2017-209458 29352014 PMC5909740

[64] Markle-Reid M, Weir R, Browne G, Roberts J, Gafni A, Henderson S. Health promotion for frail older home care clients. J Adv Nurs. 2006;54(3):381-395. doi:10.1111/j.1365-2648.2006.03817.x 16629922

[65] Parry SW, Bamford C, Deary V, . Cognitive-behavioural therapy-based intervention to reduce fear of falling in older people: therapy development and randomised controlled trial—the Strategies for Increasing Independence, Confidence and Energy (STRIDE) study. Health Technol Assess. 2016;20(56):1-206. doi:10.3310/hta20560 27480813 PMC4983706

[66] Giovagnoli AR, Manfredi V, Schifano L, Paterlini C, Parente A, Tagliavini F. Combining drug and music therapy in patients with moderate Alzheimer’s disease: a randomized study. Neurol Sci. 2018;39(6):1021-1028. doi:10.1007/s10072-018-3316-3 29550981

[67] Jansons P, Robins L, O’Brien L, Haines T. Gym-based exercise and home-based exercise with telephone support have similar outcomes when used as maintenance programs in adults with chronic health conditions: a randomised trial. J Physiother. 2017;63(3):154-160. doi:10.1016/j.jphys.2017.05.018 28655559

[68] Morgenstern LB, Adelman EE, Hughes R, Wing JJ, Lisabeth LD. The Women Independently Living Alone with a Medical Alert device (WILMA) trial. Transl Stroke Res. 2015;6(5):355-360. doi:10.1007/s12975-015-0411-0 26031786 PMC4560987

[69] Yap AF, Kwan YH, Tan CS, Ibrahim S, Ang SB. Rhythm-centred music making in community living elderly: a randomized pilot study. BMC Complement Altern Med. 2017;17(1):1. doi:10.1186/s12906-017-1825-x 28615007 PMC5470187

[70] Banks MR, Banks WA. The effects of animal-assisted therapy on loneliness in an elderly population in long-term care facilities. J Gerontol A Biol Sci Med Sci. 2002;57(7):M428-M432. doi:10.1093/gerona/57.7.M428 12084804

[71] Banks MR, Banks WA. The effects of group and individual animal-assisted therapy on loneliness in residents of long-term care facilities. Anthrozoös. 2005;18(4):396-408. doi:10.2752/089279305785593983

[72] Banks MR, Willoughby LM, Banks WA. Animal-assisted therapy and loneliness in nursing homes: use of robotic versus living dogs. J Am Med Dir Assoc. 2008;9(3):173-177. doi:10.1016/j.jamda.2007.11.007 18294600

[73] Sollami A, Gianferrari E, Alfieri M, Artioli G, Taffurelli C. Pet therapy: an effective strategy to care for the elderly? an experimental study in a nursing home. Acta Biomed. 2017;88(1S):25-31. doi:10.23750/abm.v88i1-S.6281 28327492 PMC10548069

[74] Robinson H, Macdonald B, Kerse N, Broadbent E. The psychosocial effects of a companion robot: a randomized controlled trial. J Am Med Dir Assoc. 2013;14(9):661-667. doi:10.1016/j.jamda.2013.02.007 23545466

[75] Jessen J, Cardiello F, Baun MM. Avian companionship in alleviation of depression, loneliness, and low morale of older adults in skilled rehabilitation units. Psychol Rep. 1996;78(1):339-348. doi:10.2466/pr0.1996.78.1.339 8839325

[76] Gustafson DH, Gustafson DH, Cody OJ, Chih MY, Johnston DC, Asthana S. Pilot test of a computer-based system to help family caregivers of dementia patients. J Alzheimers Dis. 2019;70(2):541-552. doi:10.3233/JAD-190052 31256126

[77] Saito T, Kai I, Takizawa A. Effects of a program to prevent social isolation on loneliness, depression, and subjective well-being of older adults: a randomized trial among older migrants in Japan. Arch Gerontol Geriatr. 2012;55(3):539-547. doi:10.1016/j.archger.2012.04.002 22564362

[78] Tse MMY, Vong SK, Ho SSK. The effectiveness of an integrated pain management program for older persons and staff in nursing homes. Arch Gerontol Geriatr. 2012;54(2):e203-e212. doi:10.1016/j.archger.2011.04.015 21592596

[79] Tse MMY, Ho SSK. Pain management for older persons living in nursing homes: a pilot study. Pain Manag Nurs. 2013;14(2):e10-e21. doi:10.1016/j.pmn.2011.01.004 23688367

[80] Tse MMY, Yeung SSY, Lee PH, Ng SSM. Effects of a peer-led pain management program for nursing home residents with chronic pain: a pilot study. Pain Med. 2016;17(9):1648-1657. doi:10.1093/pm/pnv121 26893112

[81] Chow AYM, Caserta M, Lund D, . Dual-Process Bereavement Group Intervention (DPBGI) for widowed older adults. Gerontologist. 2019;59(5):983-994. doi:10.1093/geront/gny095 30137473

[82] Cohen-Mansfield J, Hazan H, Lerman Y, Shalom V, Birkenfeld S, Cohen R. Efficacy of the I-SOCIAL intervention for loneliness in old age: lessons from a randomized controlled trial. J Psychiatr Res. 2018;99:69-75. doi:10.1016/j.jpsychires.2018.01.014 29407289

[83] Mountain G, Windle G, Hind D, . A preventative lifestyle intervention for older adults (lifestyle matters): a randomised controlled trial. Age Ageing. 2017;46(4):627-634. doi:10.1093/ageing/afx021 28338849 PMC5860501

[84] Kremers IP, Steverink N, Albersnagel FA, Slaets JPJ. Improved self-management ability and well-being in older women after a short group intervention. Aging Ment Health. 2006;10(5):476-484. doi:10.1080/13607860600841206 16938683

[85] Routasalo PE, Tilvis RS, Kautiainen H, Pitkala KH. Effects of psychosocial group rehabilitation on social functioning, loneliness and well-being of lonely, older people: randomized controlled trial. J Adv Nurs. 2009;65(2):297-305. doi:10.1111/j.1365-2648.2008.04837.x 19054177

[86] Alaviani M, Khosravan S, Alami A, Moshki M. The effect of a multi-strategy program on developing social behaviors based on Pender’s Health Promotion Model to prevent loneliness of old women referred to Gonabad urban health centers. Int J Community Based Nurs Midwifery. 2015;3(2):132-140. 26005693 PMC4441350

[87] Ehlers DK, Daugherty AM, Burzynska AZ, . Regional brain volumes moderate, but do not mediate, the effects of group-based exercise training on reductions in loneliness in older adults. Front Aging Neurosci. 2017;9:110. doi:10.3389/fnagi.2017.00110 28487648 PMC5403947

[88] Baez M, Khaghani Far I, Ibarra F, Ferron M, Didino D, Casati F. Effects of online group exercises for older adults on physical, psychological and social wellbeing: a randomized pilot trial. PeerJ. 2017;5:e3150. doi:10.7717/peerj.3150 28392983 PMC5384569

[89] Jones CA, Siever J, Knuff K, . Walk, Talk and Listen: a pilot randomised controlled trial targeting functional fitness and loneliness in older adults with hearing loss. BMJ Open. 2019;9(4):e026169. doi:10.1136/bmjopen-2018-026169 30987987 PMC6500300

[90] Chan AWK, Yu DSF, Choi KC. Effects of tai chi qigong on psychosocial well-being among hidden elderly, using elderly neighborhood volunteer approach: a pilot randomized controlled trial. Clin Interv Aging. 2017;12:85-96. doi:10.2147/CIA.S124604 28115837 PMC5221552

[91] Wang DS. Feasibility of a yoga intervention for enhancing the mental well-being and physical functioning of older adults living in the community. Act Adaptation Aging. 2010;34(2):85-97. doi:10.1080/01924781003773559

[92] Johnson JK, Stewart AL, Acree M, . A community choir intervention to promote well-being among diverse older adults: results from the Community of Voices Trial. J Gerontol B Psychol Sci Soc Sci. 2020;75(3):549-559. doi:10.1093/geronb/gby132 30412233 PMC7328053

[93] de Craen AJM, Gussekloo J, Blauw GJ, Willems CG, Westendorp RGJ. Randomised controlled trial of unsolicited occupational therapy in community-dwelling elderly people: the LOTIS trial. PLoS Clin Trials. 2006;1(1):e2. doi:10.1371/journal.pctr.0010002 16871324 PMC1488896

[94] Larsson E, Padyab M, Larsson-Lund M, Nilsson I. Effects of a social internet-based intervention programme for older adults: an explorative randomised crossover study. Br J Occup Ther. 2016;79(10):629-636. doi:10.1177/0308022616641701

[95] Chiang KJ, Chu H, Chang HJ, . The effects of reminiscence therapy on psychological well-being, depression, and loneliness among the institutionalized aged. Int J Geriatr Psychiatry. 2010;25(4):380-388. doi:10.1002/gps.2350 19697299

[96] Westerhof GJ, Korte J, Eshuis S, Bohlmeijer ET. Precious memories: a randomized controlled trial on the effects of an autobiographical memory intervention delivered by trained volunteers in residential care homes. Aging Ment Health. 2018;22(11):1494-1501. doi:10.1080/13607863.2017.1376311 28929782

[97] Moieni M, Seeman TE, Robles TF, . Generativity and social well-being in older women: expectations regarding aging matter. J Gerontol B Psychol Sci Soc Sci. 2021;76(2):289-294. doi:10.1093/geronb/gbaa022 32064530 PMC7813180

[98] Rook KS, Sorkin DH. Fostering social ties through a volunteer role: implications for older-adults’ psychological health. Int J Aging Hum Dev. 2003;57(4):313-337. doi:10.2190/NBBN-EU3H-4Q1N-UXHR 15195981

[99] Mountain GA, Hind D, Gossage-Worrall R, . ‘Putting Life in Years’ (PLINY) telephone friendship groups research study: pilot randomised controlled trial. Trials. 2014;15:141. doi:10.1186/1745-6215-15-141 24758530 PMC4022155

[100] Andersson L. Intervention against loneliness in a group of elderly women: a process evaluation. Hum Relat. 1984;37(4):295-310. doi:10.1177/001872678403700402 3992279

[101] Hartke RJ, King RB. Telephone group intervention for older stroke caregivers. Top Stroke Rehabil. 2003;9(4):65-81. doi:10.1310/RX0A-6E2Y-BU8J-W0VL 14523701

[102] Charlesworth G, Shepstone L, Wilson E, Thalanany M, Mugford M, Poland F. Does befriending by trained lay workers improve psychological well-being and quality of life for carers of people with dementia, and at what cost? a randomised controlled trial. Health Technol Assess. 2008;12(4):1-78. doi:10.3310/hta12040 18284895

[103] Bickmore TW, Caruso L, Clough-Gorr K, Heeren T. “It’s just like you talk to a friend” relational agents for older adults. Interact Comput. 2005;17(6):711-735. doi:10.1016/j.intcom.2005.09.002

[104] Czaja SJ, Boot WR, Charness N, Rogers WA, Sharit J. Improving social support for older adults through technology: findings from the PRISM randomized controlled trial. Gerontologist. 2018;58(3):467-477. doi:10.1093/geront/gnw249 28201730 PMC5946917

[105] Dodge HH, Zhu J, Mattek N, . Web-enabled conversational interactions as a method to improve cognitive functions: results of a 6-week randomized controlled trial. Alzheimers Dement (N Y). 2015;1(1):1-12. doi:10.1016/j.trci.2015.01.001 26203461 PMC4507295

[106] Sidner CL, Bickmore T, Nooraie B, . Creating new technologies for companionable agents to support isolated older adults. ACM Trans Interact Intell Syst. 2018;8(3):1-27. doi:10.1145/3213050

[107] Slegers K, van Boxtel M, Jolles J. Effects of computer training and internet usage on cognitive abilities in older adults: a randomized controlled study. Aging Clin Exp Res. 2009;21(1):43-54. doi:10.1007/BF03324898 19225269

[108] Tsai HH, Tsai YF. Changes in depressive symptoms, social support, and loneliness over 1 year after a minimum 3-month videoconference program for older nursing home residents. J Med Internet Res. 2011;13(4):e93. doi:10.2196/jmir.1678 22086660 PMC3222194

[109] Tsai HH, Cheng CY, Shieh WY, Chang YC. Effects of a smartphone-based videoconferencing program for older nursing home residents on depression, loneliness, and quality of life: a quasi-experimental study. BMC Geriatr. 2020;20(1):27. doi:10.1186/s12877-020-1426-2 31992217 PMC6986028

[110] Jarvis MA, Padmanabhanunni A, Chipps J. An evaluation of a low-intensity cognitive behavioral therapy mhealth-supported intervention to reduce loneliness in older people. Int J Environ Res Public Health. 2019;16(7):1305. doi:10.3390/ijerph16071305 30979042 PMC6480633

[111] Theeke LA, Mallow JA, Moore J, McBurney A, Rellick S, VanGilder R. Effectiveness of LISTEN on loneliness, neuroimmunological stress response, psychosocial functioning, quality of life, and physical health measures of chronic illness. Int J Nurs Sci. 2016;3(3):242-251. doi:10.1016/j.ijnss.2016.08.004 29082303 PMC5656260

[112] Nelson CJ, Saracino RM, Roth AJ, . Cancer and Aging: Reflections for Elders (CARE): a pilot randomized controlled trial of a psychotherapy intervention for older adults with cancer. Psychooncology. 2019;28(1):39-47. doi:10.1002/pon.4907 30296337 PMC6476184

[113] Hawkley LC, Cacioppo JT. Loneliness matters: a theoretical and empirical review of consequences and mechanisms. Ann Behav Med. 2010;40(2):218-227. doi:10.1007/s12160-010-9210-8 20652462 PMC3874845

[114] Jarvis MA, Padmanabhanunni A, Balakrishna Y, Chipps J. The effectiveness of interventions addressing loneliness in older persons: an umbrella review. Int J Africa Nurs Sci. 2020;12:100177. doi:10.1016/j.ijans.2019.100177

[115] Wise JB. Social cognitive theory: a framework for therapeutic recreation practice. Ther Recreation J. 2002;36(4):335-351.

[116] Costa-Cordella S, Arevalo-Romero C, Parada FJ, Rossi A. Social support and cognition: a systematic review. Front Psychol. 2021;12:637060. doi:10.3389/fpsyg.2021.637060 33708164 PMC7941073

[117] Beadle JN, de la Vega CE. Impact of aging on empathy: review of psychological and neural mechanisms. Front Psychiatry. 2019;10:331. doi:10.3389/fpsyt.2019.00331 31244684 PMC6580149

[118] Cattan M, White M, Bond J, Learmouth A. Preventing social isolation and loneliness among older people: a systematic review of health promotion interventions. Ageing Soc. 2005;25(1):41-67. doi:10.1017/S0144686X04002594 27736564

[119] Fakoya OA, McCorry NK, Donnelly M. Loneliness and social isolation interventions for older adults: a scoping review of reviews. BMC Public Health. 2020;20(1):129. doi:10.1186/s12889-020-8251-6 32054474 PMC7020371

[120] Stuart A, Stevenson C, Koschate M, Cohen J, Levine M. ‘Oh no, not a group!’ The factors that lonely or isolated people report as barriers to joining groups for health and well-being. Br J Health Psychol. 2022;27(1):179-193. doi:10.1111/bjhp.12536 34028949

[121] Hall JA. How many hours does it take to make a friend?. J Soc Pers Relat. 2019;36(4):1278-1296. doi:10.1177/0265407518761225

[122] Prieto-Flores ME, Forjaz MJ, Fernandez-Mayoralas G, Rojo-Perez F, Martinez-Martin P. Factors associated with loneliness of noninstitutionalized and institutionalized older adults. J Aging Health. 2011;23(1):177-194. doi:10.1177/0898264310382658 20881107

[123] Bethell J, Aelick K, Babineau J, . Social connection in long-term care homes: a scoping review of published research on the mental health impacts and potential strategies during COVID-19. J Am Med Dir Assoc. 2021;22(2):228-237.e25. doi:10.1016/j.jamda.2020.11.025 33347846 PMC9186333

[124] Handley M, Bunn F, Dunn V, Hill C, Goodman C. Effectiveness and sustainability of volunteering with older people living in care homes: a mixed methods systematic review. Health Soc Care Community. 2022;30(3):836-855. doi:10.1111/hsc.13576 34558761

[125] Charpentier M, Kirouac L. Experiences of loneliness among older people living alone: a qualitative study in Quebec (Canada). Ageing Soc. 2021:1-22. doi:10.1017/S0144686X21000349

[126] Machielse A. The heterogeneity of socially isolated older adults: a social isolation typology. J Gerontol Soc Work. 2015;58(4):338-356. doi:10.1080/01634372.2015.1007258 25588026

[127] Gilbey A, Tani K. Companion animals and loneliness: a systematic review of quantitative studies. Anthrozoös. 2015;28(2):181-137. doi:10.1080/08927936.2015.11435396

[128] Jain B, Syed S, Hafford-Letchfield T, O’Farrell-Pearce S. Dog-assisted interventions and outcomes for older adults in residential long-term care facilities: a systematic review and meta-analysis. Int J Older People Nurs. 2020;15(3):e12320. doi:10.1111/opn.12320 32394594

[129] Chen YR, Schulz PJ. The effect of information communication technology interventions on reducing social isolation in the elderly: a systematic review. J Med Internet Res. 2016;18(1):e18. doi:10.2196/jmir.4596 26822073 PMC4751336

[130] Choi M, Kong S, Jung D. Computer and internet interventions for loneliness and depression in older adults: a meta-analysis. Healthc Inform Res. 2012;18(3):191-198. doi:10.4258/hir.2012.18.3.191 23115742 PMC3483477

[131] Hagan R, Manktelow R, Taylor BJ, Mallett J. Reducing loneliness amongst older people: a systematic search and narrative review. Aging Ment Health. 2014;18(6):683-693. doi:10.1080/13607863.2013.875122 24437736

[132] Cacioppo JT, Hawkley LC. People thinking about people: the vicious cycle of being a social outcast in one’s own mind. In: Williams KD, Forgas JP, von Hippel W, eds. The Social Outcast: Ostracism, Social Exclusion, Rejection, and Bullying. Psychology Press; 2005:91-108. Accessed March 2, 2022. https://psycnet.apa.org/record/2005-13813-006

[133] Milligan C, Dowrick C, Payne S, . Men’s sheds and other gendered interventions for older men: improving health and wellbeing—a systematic review and scoping of the evidence base. 2013. Accessed September 7, 2022. https://www.ageing.ox.ac.uk/publications/view/450

