# Supplementary material for: Interventions Associated With Reduced Loneliness and Social Isolation in Older Adults: A Systematic Review and Meta-analysis
Source: JAMA Netw Open. 2022 Oct 17;5(10):e2236676. doi: 10.1001/jamanetworkopen.2022.36676 (PMC9577679; doi:10.1001/jamanetworkopen.2022.36676)
Supplement: Supplement. — eTable 1. Search Strategy With Each Corresponding Database eTable 2. Cohen κ for Reviewed Abstracts eTable 3. Estimating Methods for Cohen d (Effect Sizes) eReferences eTable 4. Intervention and Control Group Characteristics of Included Studies eTable 5. Reasons for Exclusion From Meta-analysis eTable 6. Meta-analyses by Intervention and Sensitivity Analyses eTable 7. GRADE Table of Included Studies eTable 8. Risk of Bias Table of Included Studies eFigure 1. Funnel Plot Analysis of Studies Included in Meta-analysis eFigure 2. Risk of Bias Assessment of Studies Included in the Systematic Review [file jamanetwopen-e2236676-s001.pdf]

Supplemental Online Content

Hoang P, King JA, Moore S, et al. Interventions associated with reduced loneliness and social isolation in older adults: a systematic review and meta-analysis. *JAMA Netw Open.* 2022;5(10):e2236676.
doi:10.1001/jamanetworkopen.2022.36676

eTable 1. Search Strategy
eTable 2. Cohen κ for Reviewed Abstracts
eTable 3. Estimating Methods for Cohen *d*
eReferences
eTable 4. Intervention and Control Group Characteristics of Included Studies
eTable 5. Reasons for Exclusion From Meta-analysis
eTable 6. Meta-analyses by Intervention and Sensitivity Analyses
eTable 7. GRADE Table of Included Studies
eTable 8. Risk of Bias Table of Included Studies
eFigure 1. Funnel Plot Analysis of Studies Included in Meta-analysis
eFigure 2. Risk of Bias Assessment of Studies Included in the Systematic Review

This supplemental material has been provided by the authors to give readers additional information about their work.

© 2022 Hoang P et al. *JAMA Network Open.*

eTable 1. Search Strategy with for Each Corresponding Database

Database	Concept 1: older adult	Concept 2: social isolation OR loneliness	Concept 3: social intervention	Concept 4: technology	Concept 5: music therapy
OVID/Medline MeSH terms	aged/ OR "aged, 80 and over"/ OR geriatrics/	social isolation/ OR loneliness/	social support/ OR socialization/ OR intergenerational relations/ OR psychosocial support systems/	culturally appropriate technology/ OR biomedical technology/ OR educational technology/ OR internet/	music therapy/
CINAHL MeSH terms*	MH "aged" OR MH "frail elderly" OR MH "aged, 80 and over" OR MH "geriatrics" OR MH "gerontological care"	MH "social isolation" OR MH "loneliness"	(MH "social networking" OR MH "social participation" OR MH "socialization" OR MH "community programs" OR MH "intergenerational relations")	MH "educational technology"	MH "music therapy" OR MH "pet therapy"
CENTRAL (Cochrane) MeSH terms	aged/ OR geriatrics/	social isolation/ OR loneliness/	social support/ OR socialization/	educational technology/ OR internet/	music therapy/
Embase MeSH terms	aged/ OR "institutionalize d adult"/ OR geriatrics/	social isolation/ OR loneliness/	social support/ OR socialization/ OR social network/ OR psychosocial care/	educational technology/ OR internet/	music therapy/
PsychINFO MeSH terms	aging/ OR geriatrics/	social isolation/ OR loneliness/	social programs/ OR social support/ OR social network/ OR socialization/ OR intergenerational relations/ OR	internet/ OR groupware/	music therapy/ OR animal assisted therapy/

© 2022 Hoang P et al. *JAMA Network Open.*

			therapeutic social clubs/		
Web of Science			No MeSH terms available		
Scopus			No MeSH terms available		
Search terms used for all databases†	older adult OR senior citizen OR elder OR elder* OR geriatric OR gerontol* OR grandparent OR retire*	loneliness OR alone* OR social isolation OR emotional isolation	(social* OR psychosocial OR community OR intergeneration*) adj3 (support* OR intervention* OR involve* OR therap* OR participat*)	technology OR computer OR mobile OR phone	music therapy OR animal therapy

*CINAHL was searched via only MeSH terms.
†Where relevant, proximity indicators were adapted according to the specific database.
MeSH: Medical Subject Headings

© 2022 Hoang P et al. *JAMA Network Open.*

eTable 2. Cohen's kappa for reviewed abstracts

Author Group	Cohen's Kappa
P.H. and J.M.	0.5975
J.M. and S.M.	0.50649
K.R. and H.S.	0.53290
C.T. and P.H.	0.63159
P.H. and K.M./J.M.*	0.57479
P.H. and C.W./J.M.*	0.775925

*Average was taken of the two authors with P.H.

© 2022 Hoang P et al. *JAMA Network Open.*

eTable 3: Estimated methods for Cohen's *d* (effect sizes)

Study	N*		RMD	SMD	Estimated method[1]	compute.es procedure[1]	Notes
	Treatment	Control					
**Animals**							
Jessen et al,[75] 1996	20	20	+0.80	+0.25	Imputed change in SDs from mean correlation coefficient of other studies[2]	mes	
Sollami, et al,[73] 2017	14	14	–8.43	–2.95	Mean changes and SDs provided in study	mes	Two intervention groups were not separated out for analysis as variations of same intervention
Banks and Banks,[70] 2002	30	15	N/A	–0.72	*F*-value from ANCOVA provided in study	fes	
Banks, and Banks,[71] 2005	17	16	+2.81	+1.49	Mean changes and SDs provided in study	mes	Both groups received a different type of the intervention.
Banks et al,[72] 2008	25	13	N/A	–2.09	*F*-value from ANOVA provided in study	fes	Two intervention groups were not separated out for analysis as variations of same intervention
Robinson et al,[74] 2013	17	17	–7.67	–1.11	Adjusted mean changes and SDs provided in study	mes	
**Therapy**							

© 2022 Hoang P et al. *JAMA Network Open.*

Jarvis et al,[110] 2019	13	17	–2.49	–1.50	Mean changes and SDs provided in study	mes	
Theeke et al,[111] 2016	15	12	–0.81	–0.79	*F*-value from ANOVA provided in study	fes	
Parry et al,[65] 2016	151	162	+0.21	+0.10	Mean differences and 95% CIs provided. Estimated SD from 95% confidence intervals for each group[2]	mes	For De Jong De Jong Gierveld Scale
Parry et al,[65] 2016	151	162	+0.69	+0.16	Mean differences and 95% CIs provided. Estimated SD from 95% confidence intervals for each group[2]	mes	For Lubben Social Network Scale
Huang et al,[59] 2011	60	60	+1.47	+0.32	Imputed change in SDs from mean correlation coefficient of other studies[2]	mes	Excluded arm that had both cognitive behavioural therapy and Tai Chi. Social satisfaction went down in both groups, but magnitude larger in control group
Nelson et al,[112] 2019	25	23	–2.08	–0.18	SMD and associated 95% confidence intervals provided in study	N/A	
Li et al,[63] 2018	61	122	N/A	+0.41	*p*-value from multilevel model provided in study	pes	Exact *p*-value not provided, so upper limit used
**Combined**							
Boen et al,[57] 2012	37	52	+0.17	+0.12	SMD provided in study	des	

© 2022 Hoang P et al. *JAMA Network Open.*

Li et al,[60] 2018	61	122	N/A	+0.41	*p*-value from mixed effect modelling provided in study	pes	Exact *p*-value not provided, so upper limit used
Huang et al,[59] 2011	56	60	N/A	+0.32	Imputed change in SDs from mean correlation coefficient of other studies[2]	mes	
Gustafson et al,[76] 2019	14	11	–0.19	–0.54	SMD provided in study	des	No loneliness improvement in treatment, just increase in controls
Tse et al,[108] 2012	296	239	–3.59	–0.32	Mean changes and SDs provided in study	mes	
Tse et al,[109] 2013	48	42	–13.10	–0.72	*p*-value from independent-sample *t* test provided in study	pes	Exact *p*-value not provided, so upper limit used
Saito et al,[77] 2012	20	37	–1.40	–0.73	*p*-value from linear mixed model provided in study	pes	
Markle-Reid et al,[64] 2006	120	122	–5.26	–0.35	Mean changes and SDs provided in study	mes	
Kapan et al,[58] 2017	34	32	–1.30	–0.04	*p*-value from ANCOVA provided in study	pes	
Wan et al,[91] 2017	57	52	+0.20	+0.29	Mean changes and SDs provided in study	mes	No actual change in intervention group, just a decrease in control
**Counselling**							
Chow et al,[81] 2019	68	33	–0.24	–0.18	SMD provided in study	des	
Kremers et al,[84] 2006	36	62	–1.30	–0.37	Calculated change in SDs from correlation	mes	Correlation coefficients based on

© 2022 Hoang P et al. *JAMA Network Open.*

					coefficients provided in study[2]		Wilcoxon signed rank tests
Mountain et al,[83] 2017	121	117	–0.70	–0.29	*p*-value from adjusted mean difference provided in study	pes	
Routasalo et al,[85] 2009	117	118	0.00	0.00	Mean differences and 95% CIs provided. Estimated SD from 95% confidence intervals for each group[2]	mes	
Alaviani et al,[86] 2015	65	75	–13.10	–3.79	Imputed change in SDs from mean correlation coefficient of other studies[2]	mes	
Cohen-Mansfield et al,[82] 2018	35	28	–0.33	–0.24	SMD provided in study	des	
**Exercise**							
Tse et al,[78] 2012	296	239	–3.59	–0.32	Mean changes and SDs provided in study	mes	
Tse et al,[80] 2016	32	18	–6.30	–0.79	Imputed change in SDs from mean correlation coefficient of other studies[2]	mes	
Kapan et al,[58] 2017	34	32	–1.30	–0.04	*p*-value from ANCOVA provided in study	pes	
Huang et al,[59] 2011	56	60	N/A	+0.32	*F*-value from mixed effect modelling provided in study	fes	
Ehlers et al,[87] 2017	45	124	N/A	–0.19	SMD provided in study	des	SMD based on mean latent change

© 2022 Hoang P et al. *JAMA Network Open.*

Baez et al,[88] 2017	20	16	+0.91	+0.44	Raw data available for download to estimate mean changes and SDs	mes	
Wang et al,[91] 2010	7	10	+0.27	+0.30	Mean changes and SDs provided in study	mes	
Jones et al,[89] 2019	29	26	-0.60	–0.35	SMD provided in study	des	
Chan et al,[90] 2017	20	15	–1.40	–0.60	SMD provided in study	des	
Jansons et al,[67] 2017	46	39	–0.40	–0.12	Raw data available for download to estimate mean changes and SDs	mes	
Wan et al,[60] 2017	57	52	+0.20	+0.29	Mean changes and SDs provided in study	mes	No actual change in intervention group, just a decrease in control
**Music**							
Giovagnoli et al,[66] 2018	22	21	+1.17	+0.07	Adjusted mean changes and SDs provided in study	mes	
Yap et al,[69] 2017	16	15	N/A	–0.36	*p*-value from generalized linear model using generalized estimating equation provided in study	pes	
Johnson et al,[92] 2020	187	170	–2.20	–0.34	SMD provided in study	des	SMD is using estimation for Hedges' *g*
**Occupational therapy**							

© 2022 Hoang P et al. *JAMA Network Open.*

Larsson et al,[94] 2016	14	14	N/A	–1.37	*F*-value from repeated measures ANOVA provided in study	fes	
De Craen et al,[93] 2006	143	154	0.00	0.00	Mean changes and SEs (used to calculate SD) provided	mes	
**Reminiscence therapy**							
Chiang et al,[95] 2009	45	47	–7.33	–1.20	Imputed change in SDs from mean correlation coefficient of other studies[2]	mes	
Westerhof et al,[96] 2017	28	23	+0.20	+0.41	SMD provided in study	des	Both intervention and control group saw reduction in loneliness
Moieni et al, 2020[97]	38	35	N/A	–0.70	*t*-statistic provided in study	tes	
**Social**							
Hartke and King,[101] 2003	43	45	–0.98	–0.22	*t*-statistic provided in study	tes	
Rook et al,[98] 2003	20	27	–0.55	–0.13	Imputed change in SDs from mean correlation coefficient of other studies[2]	mes	Only included foster grandparent arm of the study
Mountain et al,[99] 2014	26	30	+0.60	+0.32	Estimated *p*-value from 95% CIs for the adjusted mean difference provided in study[3]	pes	
Charlesworth et al,[102] 2008	93	97	–0.17	–0.09	*p*-value from generalized linear	pes	

© 2022 Hoang P et al. *JAMA Network Open.*

					model provided in study		
MacIntyre et al,[61] 1999	12	10	+5.48	+1.02	Mean changes and SDs provided in study	mes	
Andersson et al,[100] 1985	35	22	+0.15	+0.31	Estimated SDs from *p*-values for the first and second measurements within both groups[2]	mes	
**Technology**							
Czaja et al,[104] 2018	135	120	N/A	–0.17	SMD provided in study	des	SMD is specific to the 6-month follow-up
Tsai et al,[108] 2011	27	28	–6.34	–0.96	χ2 from generalized estimating equation provided in study	chies	
Tsai et al,[109] 2020	20	20	–4.80	–1.95	χ2 from generalized estimating equation provided in study	chies	
Gustafson et al,[76] 2019	14	11	–0.19	–0.54	SMD provided in study	des	
Sidner et al,[106] 2018	26	10	N/A	+0.05	*p*-value from ANOVA provided in study	pes	Two intervention groups were not separated out for analysis as variations of technological intervention.
Slegers et al,[107] 2008	57	133	N/A	+0.03	*p*-value from repeated measures ANOVA provided in study	pes	
Morgenstern et al,[65] 2015	112	122	–0.08	–0.18	Mean differences and 95% CIs provided.	mes	

					Estimated SD from 95% confidence intervals for each group[2]		
Dodge et al,[105] 2015	41	42	–0.20	–0.18	Author provided mean differences and changes in SD	mes	
Bond et al,[62] 2010	31	31	+0.65	+1.00	SMD provided in study	des	
Wan et al,[60] 2017	57	52	+0.20	+0.29	Mean changes and SDs provided in study	mes	No actual change in intervention group, just a decrease in control
Bickmore et al,[103] 2005	8	9	N/A	+0.79	*t*-statistic provided in study	tes	Value used is for comparison of values post-intervention for both groups

RMD = raw mean difference; SMD = standardized mean difference; SD = standard deviation; SE = standard error; CI = confidence interval; ANOVA = analysis of variance; ANCOVA = analysis of covariance

*Sample sizes were derived from the number of participants that completed the trial for the most conservative estimates.

References

1. Del Re A. compute.es: Compute Effect Sizes. https://cran.r-project.org/web/packages/compute.es/index.html. Published 2020. Accessed September 19, 2021.
2. Higgins JPT, Green S (editors). *Cochrane Handbook for Systematic Reviews of Interventions Version 5.1.0* [updated March 2011]. The Cochrane Collaboration, 2011. Available from www.handbook.cochrane.org.
3. Altman DG, Bland JM. How to obtain the P value from a confidence interval. *Bmj*. 2011 Aug 8;343.

© 2022 Hoang P et al. *JAMA Network Open.*

eTable 4. Intervention and control arm characteristics of included studies

**Author/Year**	**Intervention description**	**Control description**	**Duration of intervention**
Animal assisted therapy			
Banks et al,[72] 2008	Living dog or robot dog	No animal assisted therapy	8 weeks
Banks, and Banks,[71] 2005	Animal assisted therapy once per week or three times per week	No animal assisted therapy	6 weeks
Banks and Banks,[70] 2002	Animal assisted therapy in a group setting	Animal assisted therapy on a one on one basis	6 weeks
Jessen et al,[75] 1996	A bird was placed in the room.	Routine care	10 days
Robinson et al,[74] 2013	PARO interactive robot	Bus trips around the city or an alternate activity	12 weeks
Sollami et al,[73] 2017	Animal therapy with a dog. Caregiving activities and teaching on physical interaction. Re-elaboration of the experience through recognition, images, and words.	Usual activities	8 weeks
Therapy			
Cox et al,[48] 2007	1) Individual psychotherapy<br>2) Small-group psychotherapy<br>Includes psychosocial coping, self-care, communication, health and social services, working with caregivers, quality of life, and end-of-life decisions	Standard case management	5 weeks
Jarvis et al,[110] 2019	Cognitive behavioural therapy via WhatsApp (Living in Network Connected Communities)	Usual care: generic wellness program for residents	3 months
Li et al,[63] 2018	Psychotherapy and family and community supports with health education	Health education only	6 months

© 2022 Hoang P et al. *JAMA Network Open.*

Nelson et al,[112] 2019	Psychotherapy	Social work calls	7 weeks
Parry et al,[65] 2016	Cognitive behavioural therapy	Usual care	8 weeks
Theeke et al,[111] 2016	Cognitive behavioural therapy: LISTEN intervention	Attention control: educational sessions	5 weeks
Multi-component			
Boen et al,[57] 2012	Three hour meetings on addressing psychosocial problems (depression symptoms, loneliness, isolation)	Daily activities, offered the same group activities after one year	35-38 weeks
Huang et al,[59] 2011	1) Combined: Tai Chi and CBT	1) Cognitive behavioural therapy only<br>2) No intervention	8 weeks
Joubert et al,[49] 2013	Combined: integrated management plan	No intervention	6 weeks
Kapan et al,[58] 2017	Combined: physical training and nutritional intervention	Social meetings	12 weeks
Markle-Reid et al,[64] 2006	Combined: home care and phone intervention	Usual home care	6 months
Ollongvist et al,[52] 2008	Combined: rehabilitation program (group activities, education)	Usual care	3 weeks
Saito et al,[77] 2012	Combined: group based education, cognitive, and social support program	No intervention	6 months
Tse et al,[78] 2012	Combined: physical exercise and arts and crafts	Regular care	8 weeks
Tse et al,[79] 2013	Combined: nursing intervention, physiotherapy, and horticulture	No intervention	8 weeks
Tse et al,[80] 2016	Pain management program: physical exercise, interactive teaching, education with peer volunteers	Weekly pain management program without peer volunteers	12 weeks

© 2022 Hoang P et al. *JAMA Network Open.*

Counseling			
Alaviani et al,[86] 2015	Educational program on symptoms of loneliness, social relationships, interpersonal action, and motivation/self-efficacy	NR	2 weeks
Chow et al,[81] 2019	Bereavement counseling	Conventional loss-oriented intervention	8 weeks
Cohen-Mansfield et al,[82] 2018	Individual or group counseling	No social engagement	6 months
Estebsari et al,[43] 2018	Eight sessions of restoration oriented and loss-components	Conventional loss-oriented orientation	8 weeks
Kremers et al,[84] 2006	Self-management counseling	No intervention	6 weeks
Mountain et al,[83] 2017	Lifestyle matters intervention	Standard care	4 months
Routasalo et al,[85] 2009	Psychosocial group sessions	No intervention	3 months
Exercise			
Baez et al,[88] 2017	Adapted OTAGO Exercise Program for falls prevention	Home-based program without social/individual persuasion	10 weeks
Chan et al,[90] 2017	Tai chi	Usual care	3 months
Ehlers et al,[87] 2017	Social dance program	1) Walk: walking sessions<br>2) Walk Plus: walking sessions and nutritional supplement<br>3) Strength, stretching, and stability exercise sessions	24 weeks
Jansons et al,[67] 2017	Gym-based individualized exercise program	Home-based exercise program	12 months

© 2022 Hoang P et al. *JAMA Network Open.*

Jones et al,[89] 2019	Exercise and walking	Group audiological rehabilitation only	10 weeks
McAuley, et al[50] 2000	Aerobic exercise intervention	Stretching and toning only	6 months
Tse et al,[55] 2014	Physical exercise program	No treatment	8 weeks
Wang et al,[91] 2010	Group yoga	Socialization only	4 weeks
Music therapy			
Giovagnoli et al,[66] 2018	Music therapy and acetylcholinesterase inhibitor	Acetylcholinesterase inhibitor only	24 weeks
Johnson et al,[92] 2020	Choir program	Waitlist control	6 months
Yap et al,[69] 2017	Rhythm wellness program	Waitlist control	11 weeks
Other/Miscellaneous			
De Craen et al,[93] 2006	Occupational therapy visit	Standard support from social services program	24 months
Larsson et al,[94] 2016	Client-based occupational therapy intervention process model	No intervention	3 months
Pynnönen et al,[53] 2018	Combined: option of exercise, social activity program, or personal counseling	One counseling session, usual services	6 months
Taube et al,[54] 2018	Case management	Standard care	12 months
Reminiscence therapy			
Chiang et al,[95] 2009	Reminiscence therapy	Waitlist control	8 weeks

© 2022 Hoang P et al. *JAMA Network Open.*

Moieni et al,[97] 2020	Generativity reminiscence therapy: writing on life experiences	Writing on neutral topics	6 weeks
Westerhof et al,[96] 2017	Precious memories: autobiographical memory intervention	Individual unstructured contacts with a volunteer	8 weeks
Social intervention			
Andersson et al,[100] 1985	Social group meetings	No intervention	2 months
Charlesworth et al,[102] 2008	Befriender facilitator intervention	Standard services	6 months
Hartke and King.,[101] 2003	Telephone support group	Usual care	8 weeks
Heller et al,[44] 1991	Telephone contact	No intervention	10 weeks
MacIntyre et al,[61] 1999	Friendly visitor program	No intervention	6 weeks
Mountain et al,[99] 2014	Befriend one-to-one call	No treatment	18 weeks
Rook et al,[98] 2003	Foster grandparent program	1) Participation in meals and activity programming<br>2) No intervention	2 years
Walshe et al,[56] 2016	Volunteer support intervention	Usual care	8 weeks
Technology			
Bickmore et al,[103] 2005	Embodied conversational agents	Physical activity intervention	2 months
Bond et al,[62] 2010	Behavioural and motivation strategies, and problem-solving skills over the internet	Standard diabetes care	6 months
Czaja et al,[104] 2018	PRISM software and connection to a PRISM buddy	Binder with resources	12 months

© 2022 Hoang P et al. *JAMA Network Open.*

Dodge et al,[105] 2015	Video chat with trainer interviewers	Weekly telephone calls	6 weeks
Gustafson et al,[76] 2019	CHESS system: motivation, decision making, stress reduction, and access to services	Book for family caregivers for patients with dementia	6 months
Morgenstern et al,[68] 2015	Medical assistance device	No medical alert device	90 days
Morton et al,[46] 2018	EasyPC computer platform	Care as usual	3 months
Nikitina et al,[51] 2018	GymCentral program: tailored training, group exercise, persuasion, remote monitoring under supervision of a remote coach	Gym Central program with limited contacts to the coach	
Sidner et al,[106] 2018	AlwaysOn system<br>1) Robot<br>2) Virtual agent	No intervention	30 days
Slegers et al,[107] 2008	Computer training and personal computer with internet access	1) Computer training, no intervention<br>2) No training, no intervention	12 months
Tsai et al,[108] 2011	Smartphone based videoconference program	NR	6 months
Tsai et al,[109] 2020	Videoconference interaction with family members	Usual activities	12 months
Wan et al,[60] 2017	Pedometer plus website: goal-setting, feedback, disease education, online community forum	Pedometer alone and written materials about exercise	13 weeks
White et al,[47] 2002	Small group internet training	Waitlist	22 weeks
Woodward et al,[45] 2011	Technology training	No intervention	6 months

Studies in which there are multiple arms are numbered in the control or intervention groups.

© 2022 Hoang P et al. *JAMA Network Open.*

eTable 5. Reasons for exclusion from meta-analysis

Author	Reason for Exclusion	Findings
Cox et al,[48] 2007	Mixed long-term care with another setting	No difference in intervention vs. control
Estebsari et al,[43] 2018	Insufficient quantitative data	Benefit in intervention vs. control*
Heller et al,[44] 1991	Large variation in groups; data not divided by those who completely received and did not receive the intervention	No difference in intervention vs. control
Joubert et al,[49] 2013	Insufficient quantitative data	Benefit in intervention vs. control
McAuley et al,[50] 2000	Insufficient quantitative data	No difference between intervention vs. control
Morton et al,[46] 2018	Mixed long-term care with another setting	No difference between intervention vs. control
Nikitina et al,[51] 2018	Insufficient quantitative data	No difference between intervention vs. control
Ollongvist et al,[52] 2008	Substitution methodology for drop-outs	No difference between intervention vs. control)
Pynnönen et al,[53] 2018	Intervention was selected by participants among three possible programs; this was not amenable to a single categorization. Data was not divided by type of intervention.	N/A (insufficient reporting)
Taube et al,[54] 2018	Insufficient quantitative data	No benefit in intervention vs. control
Tse et al,[55] 2014	Same data present from a separate study	No benefit in intervention vs. control
Walshe et al,[56] 2016	Insufficient quantitative data	No difference in intervention vs. control
White et al,[47] 2002	Mixed long-term care with another setting	No difference in intervention vs. control
Woodward et al,[45] 2011	Insufficient quantitative data	No difference in intervention vs. control

*****Information describes the finding of the study with respect to the outcome of interest

© 2022 Hoang P et al. *JAMA Network Open.*

eTable 6: Meta-analyses by intervention and sensitivity analyses

Intervention	Loneliness			No outliers			No combined			No active controls			Social isolation			Social support*		
	N	Cohen's *d* (95% CI)	I^2	N	Cohen's *d* (95% CI)	I^2	N	Cohen's *d* (95% CI)	I^2	N	Cohen's *d* (95% CI)	I^2	N	Cohen's *d* (95% CI)	I^2	N	Cohen's *d* (95% CI)	I^2
**Animal therapy**[70-75]																		
**Community**	2	-0.41 (-1.75, 0.92)	87%	N/A	N/A	N/A	N/A	N/A	N/A	1	0.25 (-0.37, 0.88)	N/A	N/A	N/A	N/A	N/A	N/A	N/A
**LTC**	4	-1.05 (-2.93, 0.84)	95%	N/A	N/A	N/A	N/A	N/A	N/A	3	-1.86 (-3.14, -0.59	86%	N/A	N/A	N/A	N/A	N/A	N/A
**Combined/Multi-Component interventions**[57-60,63,64,76-80]																		
**Community**	2	-0.67 (-1.13, -0.21)	0%	N/A	N/A	N/A	N/A	N/A	N/A	N/A	N/A	N/A	N/A	N/A	N/A	6	0.29 (0.15, 0.43)	0%
**LTC**	3	-0.53 (-0.86, -0.20)	57%	N/A	N/A	N/A	N/A	N/A	N/A	1	-0.32 (-0.49, -0.15)	N/A	N/A	N/A	N/A	N/A	N/A	N/A
**Counselling**[81-86]																		
**Community**	6	-0.80 (-1.96, 0.36)	97%	5	-0.19 (-0.35, -0.03)	0%	N/A	N/A	N/A	5	-0.93 (-2.32, 0.46)	97%	N/A	N/A	N/A	N/A	N/A	N/A
**LTC**	N/A	N/A	N/A	N/A	N/A	N/A	N/A	N/A	N/A	N/A	N/A	N/A	N/A	N/A	N/A	N/A	N/A	N/A
**Exercise**[57-59,67,78-80,87-91]																		
**Community**	5	-0.15 (-0.44, 0.15)	35%	N/A	N/A	N/A	N/A	N/A	N/A	2	-0.45 (-0.86, -0.03)	0%	1	-0.12 (-0.55, 0.31)	N/A	3	0.17 (-0.07, 0.41)	0%
**LTC**	3	-0.53 (-0.86, -0.20)	57%	N/A	N/A	N/A	N/A	N/A	N/A	1	-0.32 (-0.49, -0.15)	N/A	N/A	N/A	N/A	N/A	N/A	N/A
**Music**[66,69,92]																		

© 2022 Hoang P et al. *JAMA Network Open.*

**Community**	1	-0.34 (-0.55, -0.13)	N/A	N/A	N/A	N/A	N/A	N/A	N/A	N/A	N/A	N/A	2	-0.11 (-0.57, 0.35)	0%	N/A	N/A	N/A
**LTC**	N/A	N/A	N/A	N/A	N/A	N/A	N/A	N/A	N/A	N/A	N/A	N/A	N/A	N/A	N/A	N/A	N/A	N/A
**Occupational therapist guided interventions**[93,94]																		
**Community**	2	-0.63 (-1.96, 0.71)	90%	N/A	N/A	N/A	N/A	N/A	N/A	N/A	N/A	N/A	N/A	N/A	N/A	N/A	N/A	N/A
**LTC**	N/A	N/A	N/A	N/A	N/A	N/A	N/A	N/A	N/A	N/A	N/A	N/A	N/A	N/A	N/A	N/A	N/A	N/A
**Reminiscence therapy**[95-97]																		
**Community**	1	-0.70 (-1.17, -0.22)	N/A	N/A	N/A	N/A	N/A	N/A	N/A	N/A	N/A	N/A	N/A	N/A	N/A	N/A	N/A	N/A
**LTC**	2	-0.40 (-1.98, 1.17)	95%	N/A	N/A	N/A	N/A	N/A	N/A	1	-1.20 (-1.65, -0.76)	N/A	N/A	N/A	N/A	N/A	N/A	N/A
**Social**[61,98-102]																		
**Community**	5	-0.02 (-0.21, 0.17)	7%	N/A	N/A	N/A	N/A	N/A	N/A	3	0.10 (-0.27, 0.48)	43%	N/A	N/A	N/A	1	1.02 (0.13, 1.91)	N/A
**LTC**	N/A	N/A	N/A	N/A	N/A	N/A	N/A	N/A	N/A	N/A	N/A	N/A	N/A	N/A	N/A	N/A	N/A	N/A
**Technology**[60,62,68,76,94,103-109]																		
**Community**	7	-0.19 (-0.51, 0.14)	59%	6	-0.09 (-0.26, 0.07)	10%	6	-0.15 (-0.53, 0.24)	63%	4	-0.04 (-0.24, 0.17)	25%	1	-0.18 (-0.43, 0.08)	N/A	2	0.62 (-0.07, 1.31)	78%
**LTC**	2	-1.40 (-2.37,-0.44)	70%	N/A	N/A	N/A	N/A	N/A	N/A	N/A	N/A	N/A	N/A	N/A	N/A	N/A	N/A	N/A
**Therapy**[63,65,110-112]																		
**Community**	4	-0.52 (-1.21, 0.17)	83%	N/A	N/A	N/A	N/A	N/A	N/A	3	-0.46 (-1.39, 0.46)	86%	1	0.16 (-0.06, 0.38)	N/A	1	0.41 (0.10, 0.72)	N/A
**LTC**	N/A	N/A	N/A	N/A	N/A	N/A	N/A	N/A	N/A	N/A	N/A	N/A	N/A	N/A	N/A	N/A	N/A	N/A

© 2022 Hoang P et al. *JAMA Network Open.*

*a positive Cohen's *d* favors intervention

© 2022 Hoang P et al. *JAMA Network Open.*

eTable 7. GRADE table of included studies

Intervention	Sub-interventions	Number of studies[1]		GRADE of evidence[1]	
		Community	LTC	Community	LTC
Animal therapy	Living dog<br>Robot dog/seal<br>Bird	2	4	Very low	Very low
Combined/Multi-component	Tai Chi and CBT<br>Integrated management, home care, rehabilitation, pain management<br>Physical training with nutrition, arts, horticulture	2	3	Very low	Very low
Counseling	Individual or group counseling on self-management, lifestyle, bereavement, psychosocial problems	6	N/A	Very Low	N/A
Exercise	Tai chi or yoga<br>Dance<br>Physical exercise	5	3	Very low	Very low
Music therapy	Choir<br>Instruments: rhythm and melodic instruments	1	N/A	Very low	N/A
Occupational therapy	Occupational therapy<br>Case management	2	N/A	Very low	N/A
Reminiscence therapy	N/A	1	2	Very low	Very low
Social intervention	Social meetings, befriending, friendly visitor, volunteers<br>Telephone groups	5	N/A	Very low	N/A
Technology	Conversational agents<br>Therapy over internet setting<br>Computer: platforms, programs, training<br>Videoconferencing	7	2	Very low	Very low

© 2022 Hoang P et al. *JAMA Network Open.*

Therapy	Psychotherapy<br>Cognitive behavioral therapy	4	N/A	Very low	N/A

[1]Includes only studies with loneliness outcomes

© 2022 Hoang P et al. *JAMA Network Open.*

eTable 8. Risk of bias table of included studies

Author/Year	Randomization	Effect of assignment to intervention	Effect of adhering to intervention	Missing outcome data	Measurement of the outcome	Selection of reporting results	Overall risk of bias
Alaviani et al,[86] 2015	Unclear	Unclear	High	Low	High	Unclear	High
Andersson,[100] 1985	Low	High	High	High	High	High	High
Baez, M. et al,[88] 2017	Unclear	High	High	High	High	Unclear	High
Banks,[72] 2008	Low	High	High	High	High	Unclear	High
Banks, and Banks,[71] 2005	Unclear	High	High	High	High	High	High
Banks,[70] 2002	Low	Unclear	High	Low	High	Unclear	High
Bickmore et al,[103] 2005	Unclear	Unclear	High	High	High	High	High
Boen et al,[57] 2012	High	High	High	High	High	High	High
Bond et al,[62] 2010	Low	Unclear	Unclear	Low	High	High	High
Chan et al,[90] 2017	Low	High	High	High	High	High	High
Charlesworth et al,[102] 2008	Unclear	High	High	High	High	High	High
Chow et al,[81] 2019	Unclear	Unclear	Unclear	High	Unclear	High	High
Cohen-Mansfield et al,[82] 2018	Unclear	High	High	High	High	High	High
Cox et al,[48] 2007	Unclear	High	High	Low	High	High	High
Czaja et al,[104] 2018	High	Unclear	High	High	High	High	High

© 2022 Hoang P et al. *JAMA Network Open.*

De Craen et al,[93] 2006	Low	Unclear	Low	Unclears	High	High	High
Dodge et al,[105] 2015	Low	Unclear	High	Low	High	Low	High
Ehlers et al,[87] 2017	Low	High	High	High	High	High	High
Estebsari et al,[43] 2018	High	High	Unclear	High	Unclear	High	High
Giovagnoli et al,[66] 2018	Low	Unclear	High	High	High	High	High
Gustafson et al,[76] 2019	Unclear	High	High	High	High	High	High
Hartke and King,[101] 2003	High	High	High	High	High	High	High
Heller et al,[44] 1991	Unclear	Unclear	High	High	High	High	High
Huang et al,[59] 2011	Low	Unclear	High	Low	High	High	High
Jansons et al,[67] 2017	Unclear	Unclear	High	High	High	High	High
Jarvis et al,[110] 2019	Low	Unclear	High	Low	High	High	High
Jessen et al,[75] 1996	Unclear	Unclear	High	Low	High	Unclear	High
Johnson et al,[92] 2020	Unclear	Unclear	Unclear	High	Unclear	Low	Unclear
Jones et al,[89] 2019	Unclear	Unclear	High	Low	High	High	High
Joubert et al,[49] 2013	High	High	High	High	High	Unclear	High
Kapan et al,[58] 2017	Unclear	Unclear	High	Low	High	Low	High

© 2022 Hoang P et al. *JAMA Network Open.*

Kremers et al,[84] 2006	Unclear	Unclear	Unclear	High	High	Unclear	High
Larsson et al,[94] 2016	High	High	High	Low	Unclear	High	High
Li et al,[63] 2018	Unclear	Unclear	Unclear	High	Unclear	High	High
MacIntyre et al,[61] 1999	Unclear	Unclear	High	Low	High	Unclear	High
Markle-Reid et al,[64] 2006	Low	High	High	High	High	High	High
McAuley et al,[50] 2000	Unclear	Unclear	High	High	High	Unclear	High
Moieni et al,[97] 2020	Unclear	High	High	High	High	High	High
Morgenstern et al,[68] 2015	Low	High	High	High	High	High	High
Morton et al,[46] 2018	Unclear	Unclear	High	Low	High	High	High
Mountain et al,[99] 2014	Low	Unclear	Unclear	Low	High	High	High
Mountain et al,[83] 2017	Low	High	Unclear	Low	High	High	High
Nelson et al,[112] 2019	Low	Unclear	High	Unclear	High	Low	High
Nikitina et al,[51] 2018	Unclear	High	High	High	High	High	High
Ollongvist et al,[52] 2008	Unclear	High	High	High	High	High	High
Parry et al,[65] 2016	Unclear	Unclear	High	Unclear	High	High	High
Pynnönen et al,[53] 2018	Unclear	High	High	High	High	High	High
Robinson et al,[74] 2013	High	Unclear	High	High	High	Unclear	High

© 2022 Hoang P et al. *JAMA Network Open.*

Rook et al,[98] 2003	High	Unclear	High	High	High	High	High
Routasalo et al,[85] 2009	Low	Unclear	High	High	Unclear	High	High
Saito et al,[77] 2012	Unclear	Unclear	High	High	High	High	High
Sidner et al,[106] 2018	Unclear	High	High	High	High	Unclear	High
Slegers et al, 2008	Unclear	High	High	High	High	High	High
Sollami et al,[107] 2017	Low	Unclear	High	Low	High	Unclear	High
Taube et al,[54] 2018	Low	Unclear	High	High	High	High	High
Theeke et al,[111] 2016	Unclear	High	High	High	High	High	High
Tsai et al,[108] 2011	High	High	Unclear	High	Unclear	High	High
Tsai. et al,[109] 2020	Unclear	Unclear	Unclear	High	Unclear	High	High
Tse et al,[78] 2012	Unclear	Unclear	Unclear	Low	Unclear	Low	Unclear
Tse et al,[79] 2013	High	Low	Unclear	Low	Unclear	Low	High
Tse et al,[55] 2014	High	High	Unclear	Low	High	Low	High
Tse et al,[80] 2016	High	Low	Unclear	Unclear	High	Low	High
Walshe et al,[56] 2016	Low	Unclear	High	Low	High	High	High
Wan et al,[60] 2017	Low	Unclear	Low	Low	High	Low	Unclear
Wang et al,[91] 2010	High	High	High	High	High	High	High
Westerhof et al,[96] 2017	Unclear	Unclear	High	Low	High	High	High

© 2022 Hoang P et al. *JAMA Network Open.*

White et al,[47] 2002	Unclear	High	High	High	High	Unclear	High
Woodward et al,[45] 2011	Unclear	High	High	High	High	High	High
Yap et al,[69] 2017	Low	High	High	High	Unclear	High	High

© 2022 Hoang P et al. *JAMA Network Open.*

eFigure 1. Funnel plot analysis of studies included in meta-analysis
a) Animal therapy: Community

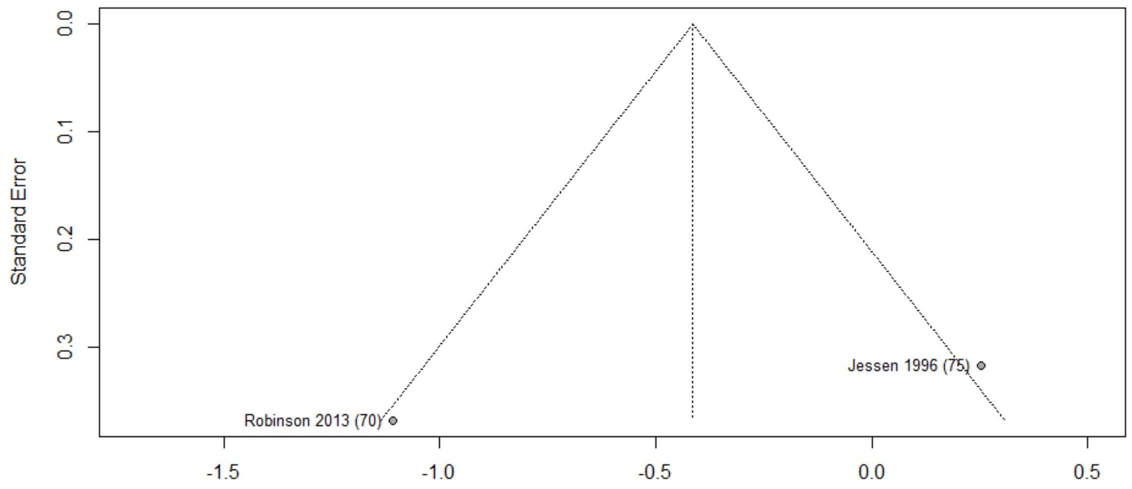

© 2022 Hoang P et al. *JAMA Network Open.*

b) Animal therapy: LTC

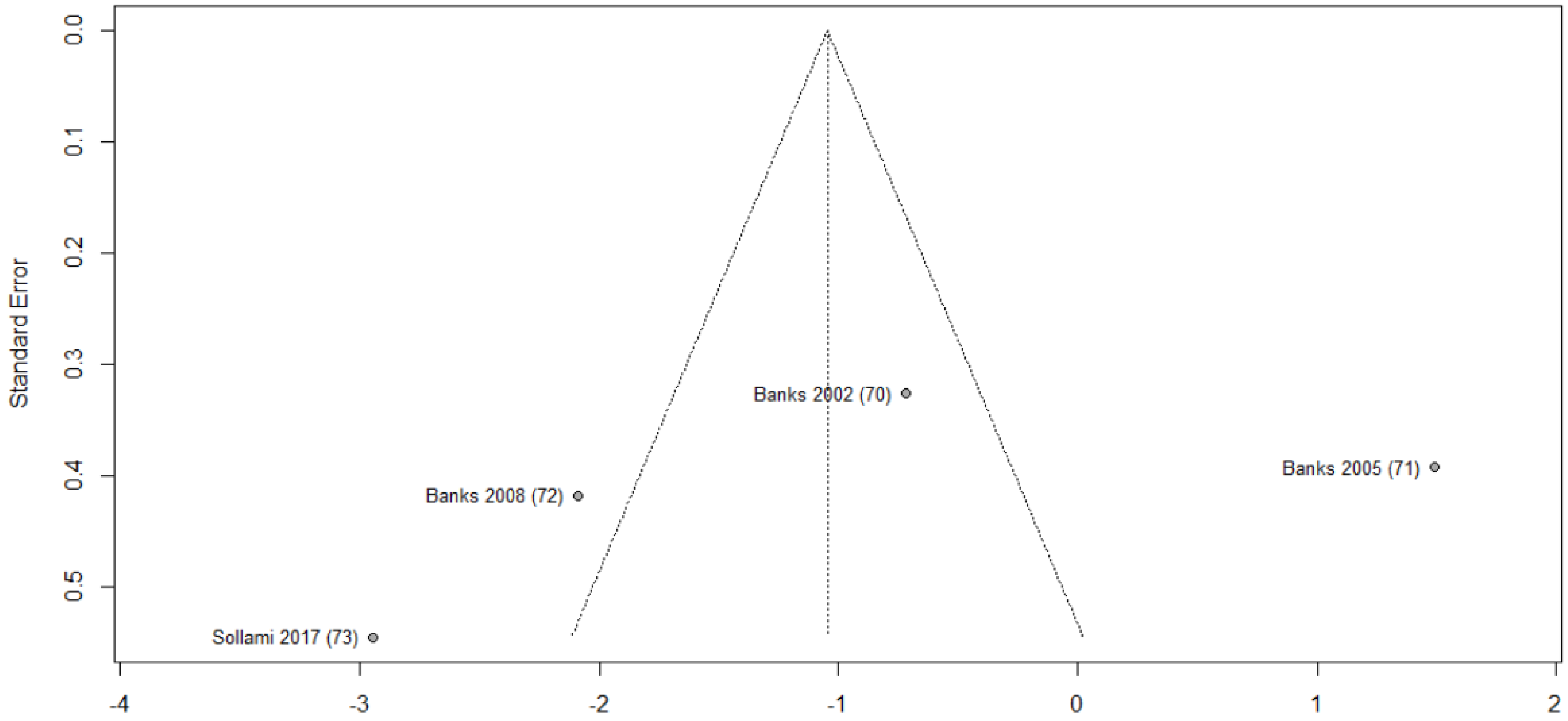

© 2022 Hoang P et al. *JAMA Network Open.*

c) Combined/Multi-component: community

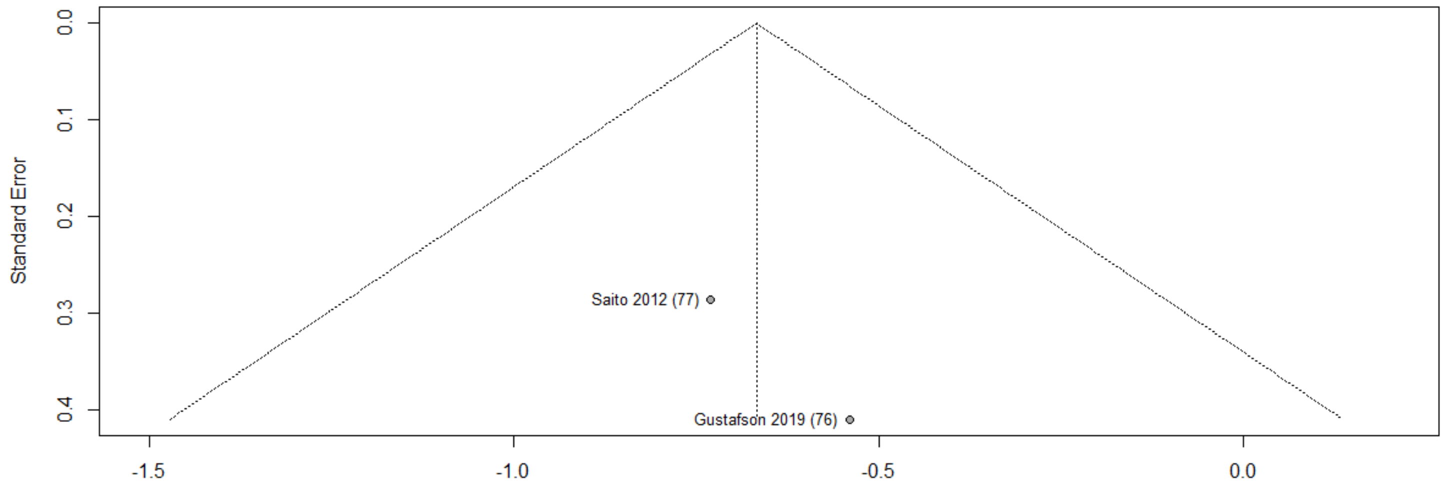

© 2022 Hoang P et al. *JAMA Network Open.*

d) Combined/Multi-component: LTC

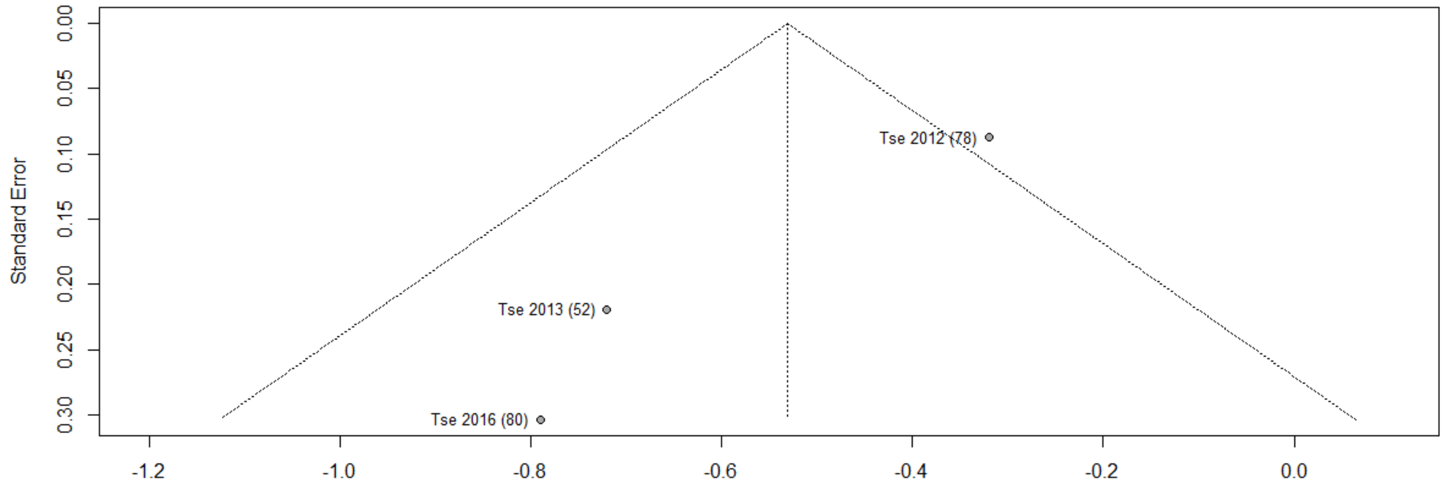

© 2022 Hoang P et al. *JAMA Network Open.*

e) Counselling: community

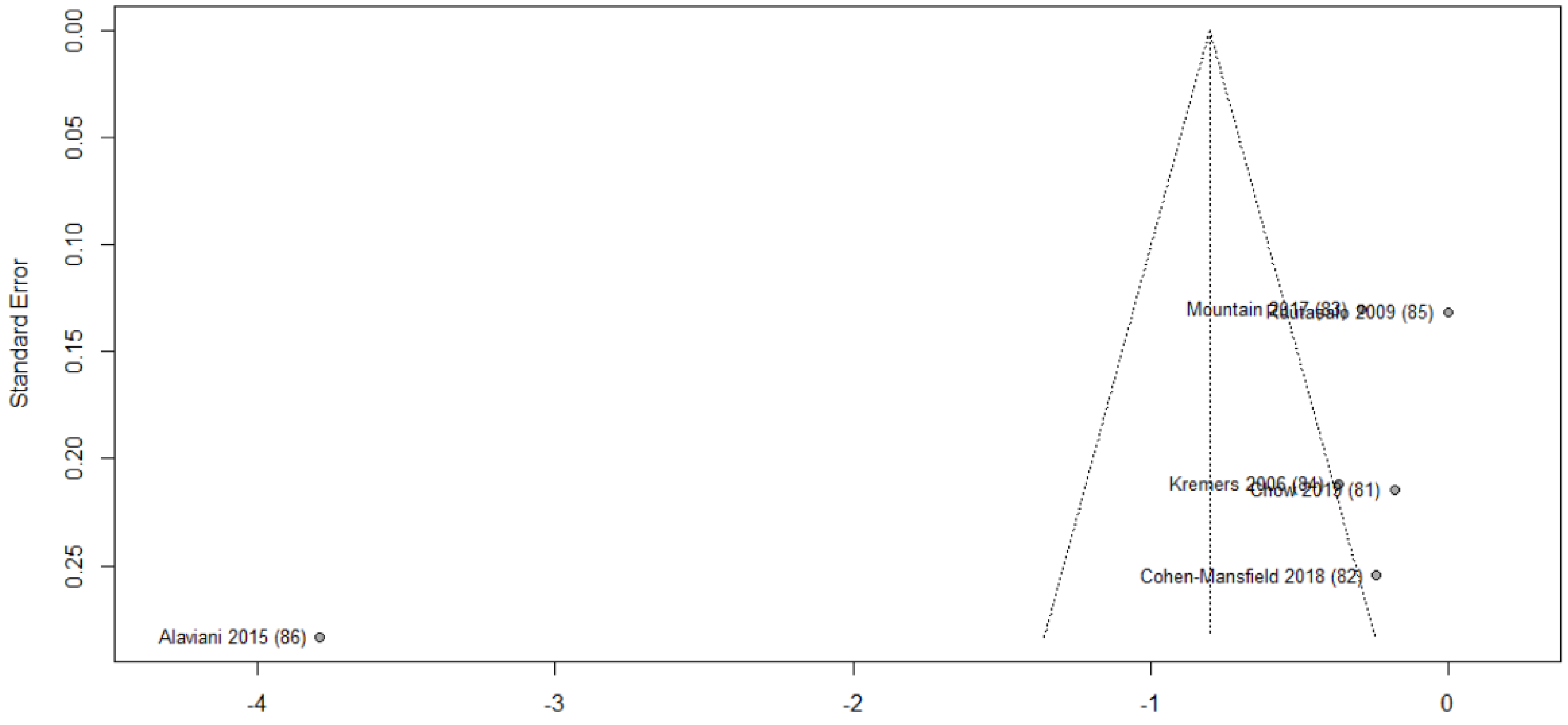

© 2022 Hoang P et al. *JAMA Network Open.*

f) Exercise: community

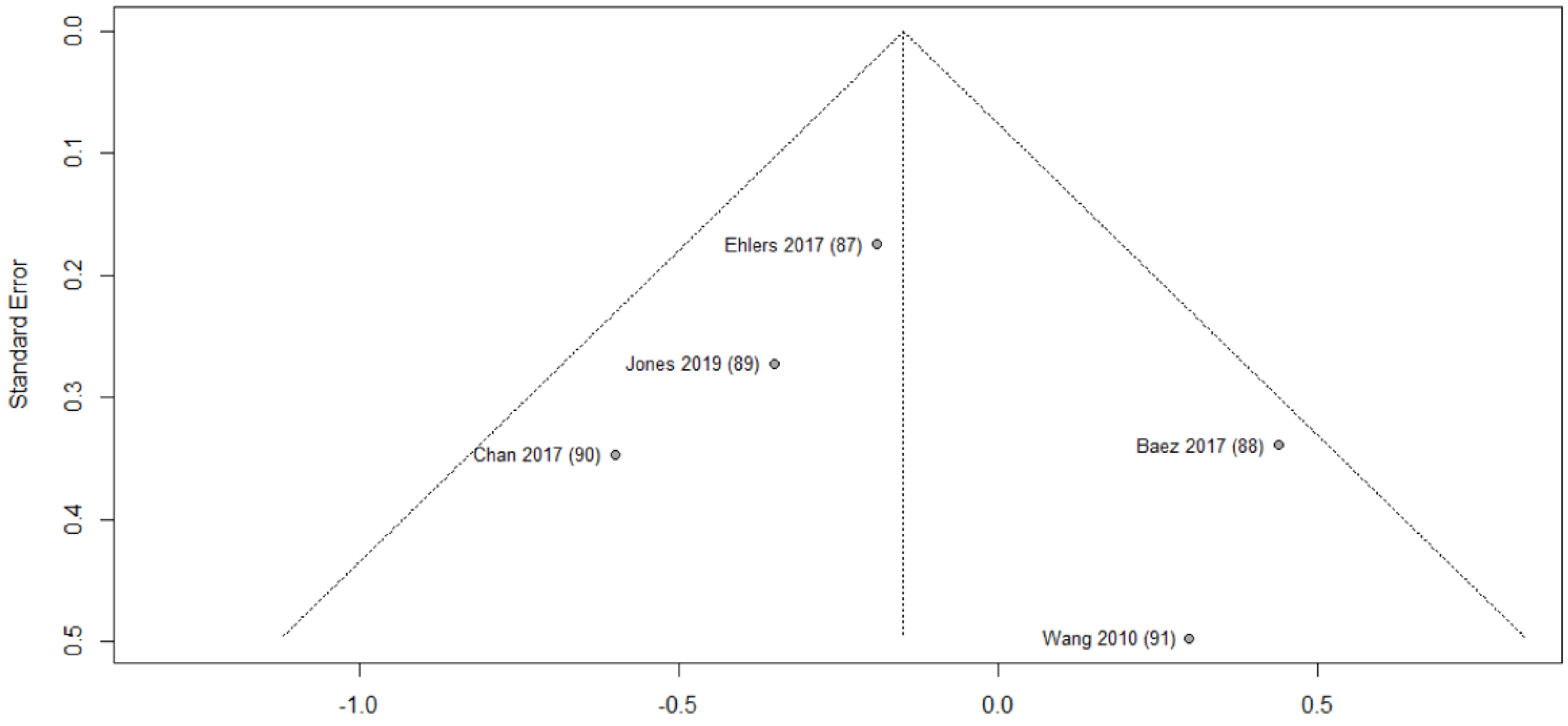

© 2022 Hoang P et al. *JAMA Network Open.*

g) Exercise: LTC

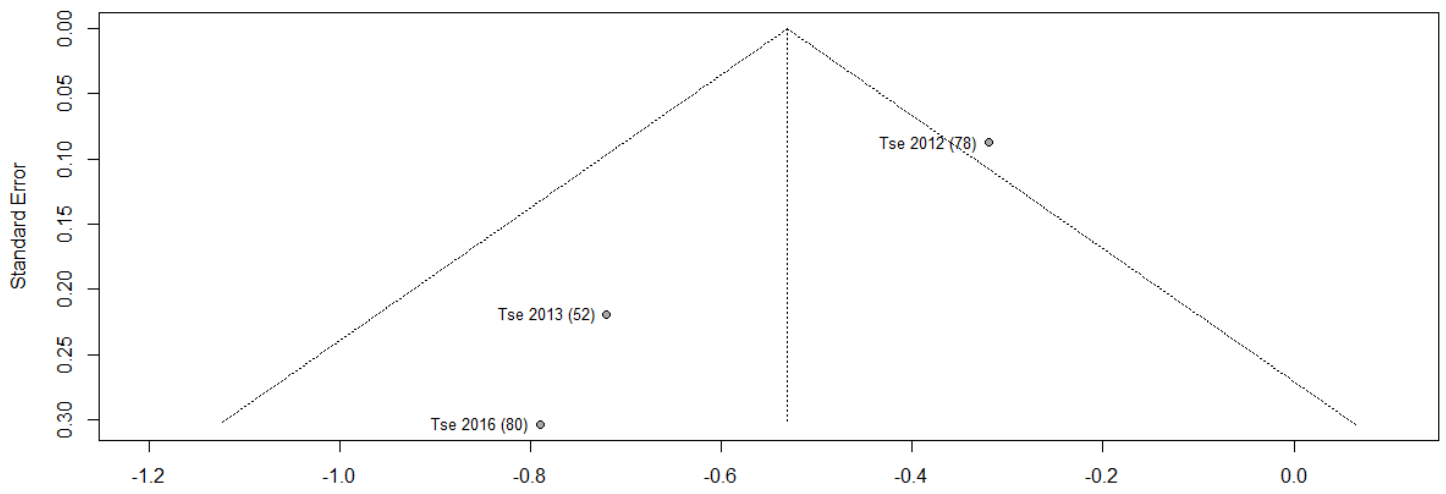

© 2022 Hoang P et al. *JAMA Network Open.*

h) Occupational therapy: community

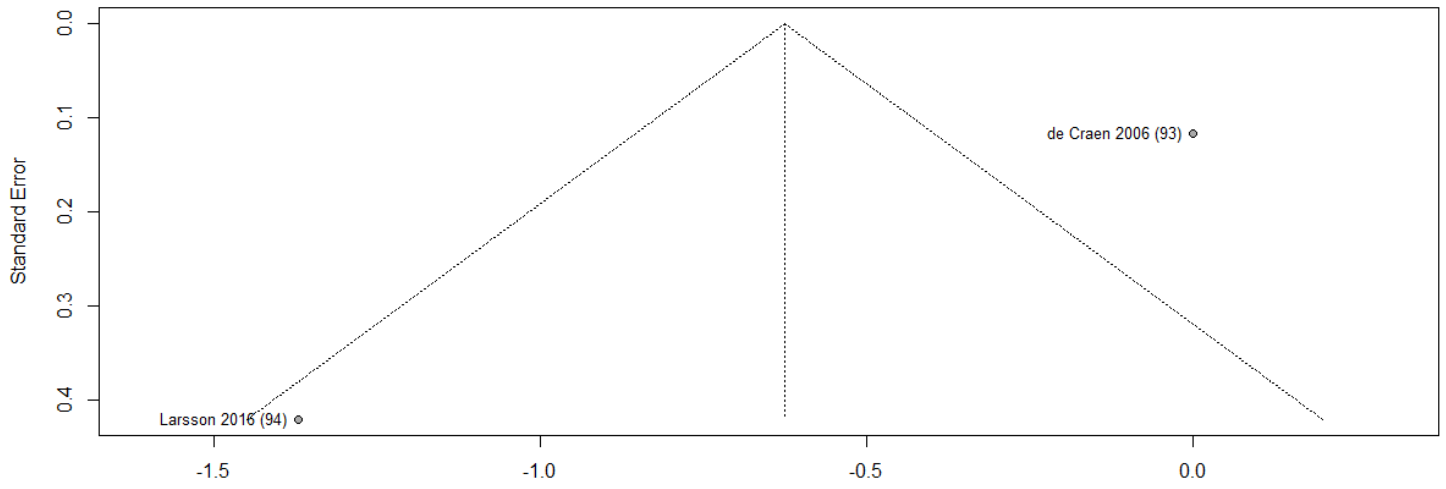

© 2022 Hoang P et al. *JAMA Network Open.*

i) Reminiscence therapy: LTC

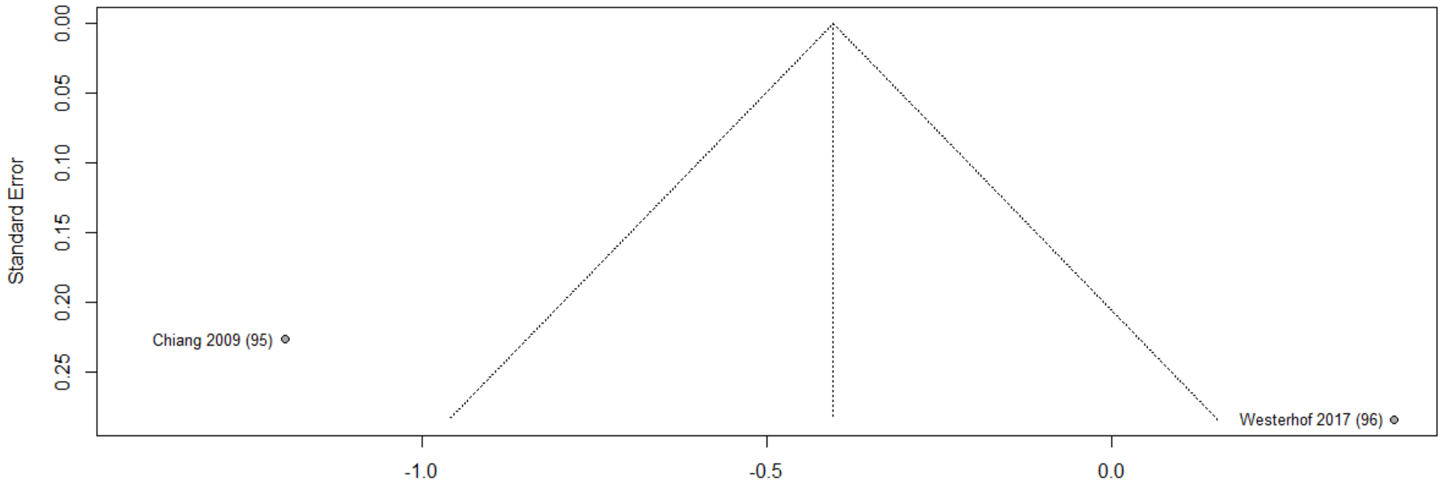

© 2022 Hoang P et al. *JAMA Network Open.*

j) Social intervention: community

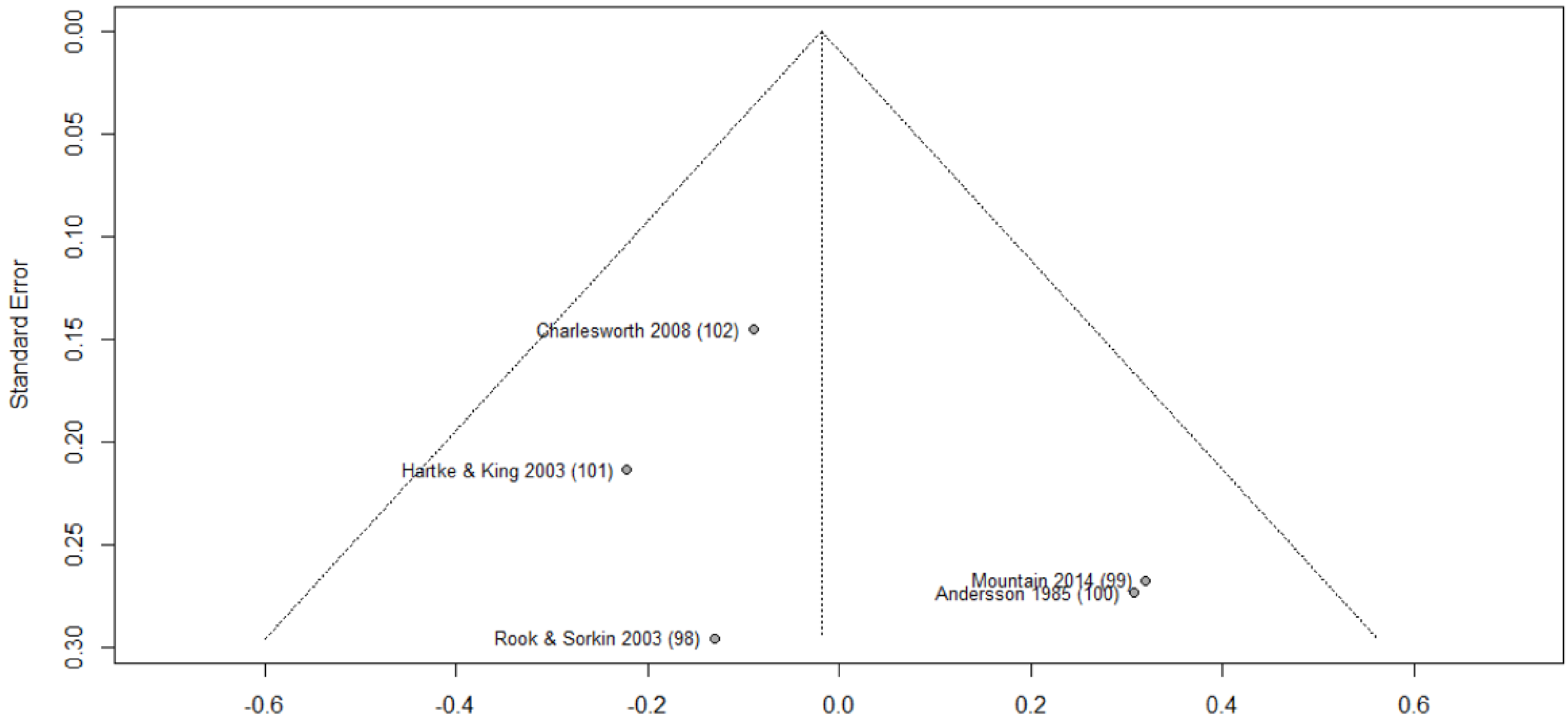

© 2022 Hoang P et al. *JAMA Network Open.*

k) Technology: community

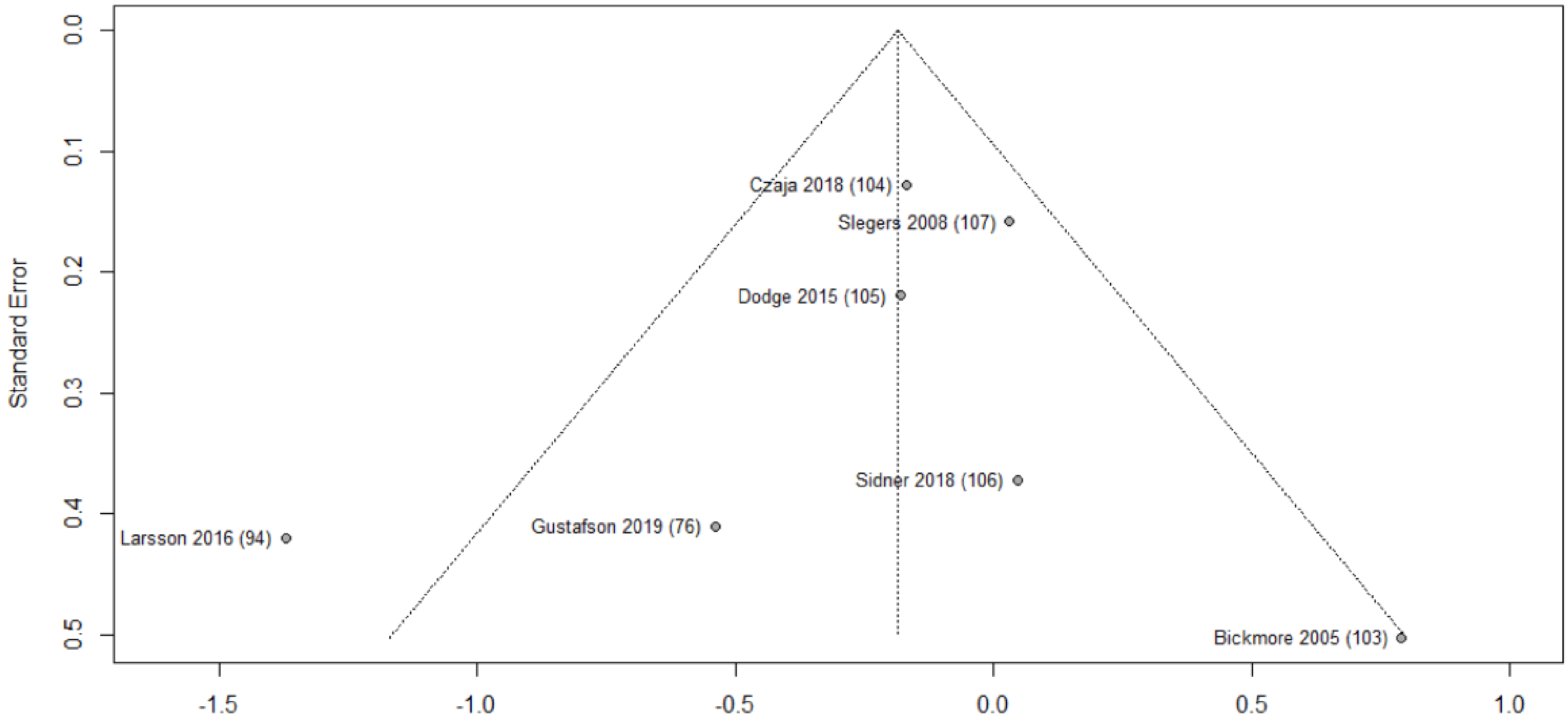

© 2022 Hoang P et al. *JAMA Network Open.*

l) Technology: LTC

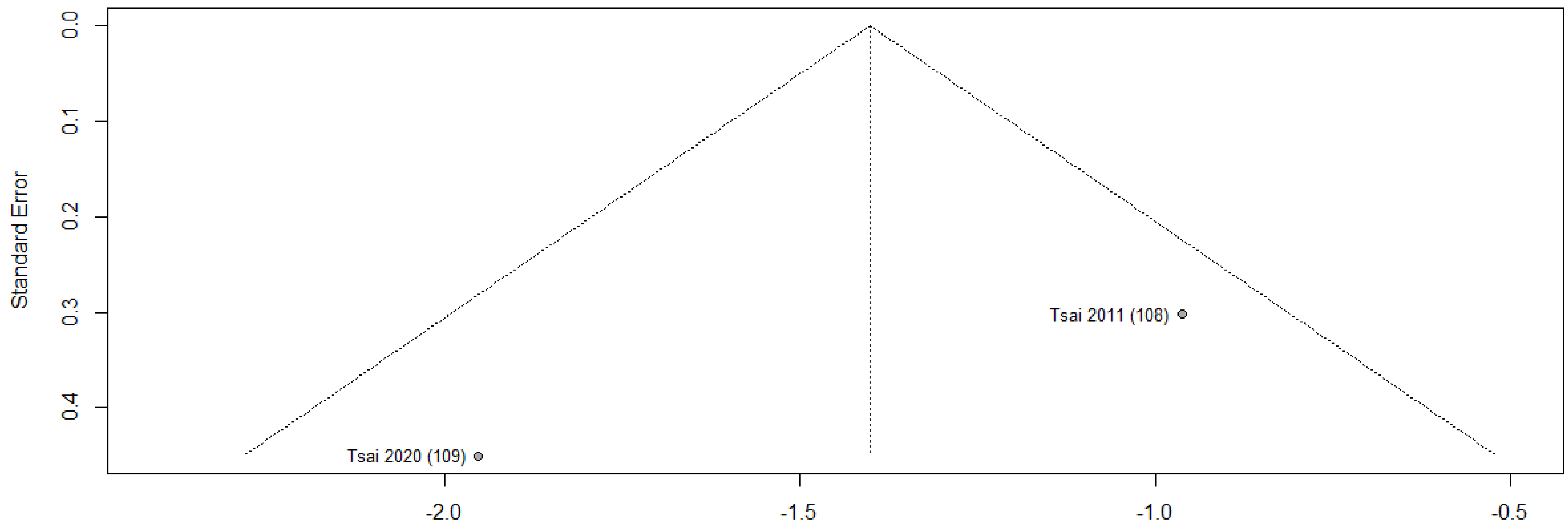

© 2022 Hoang P et al. *JAMA Network Open.*

m) Therapy: community

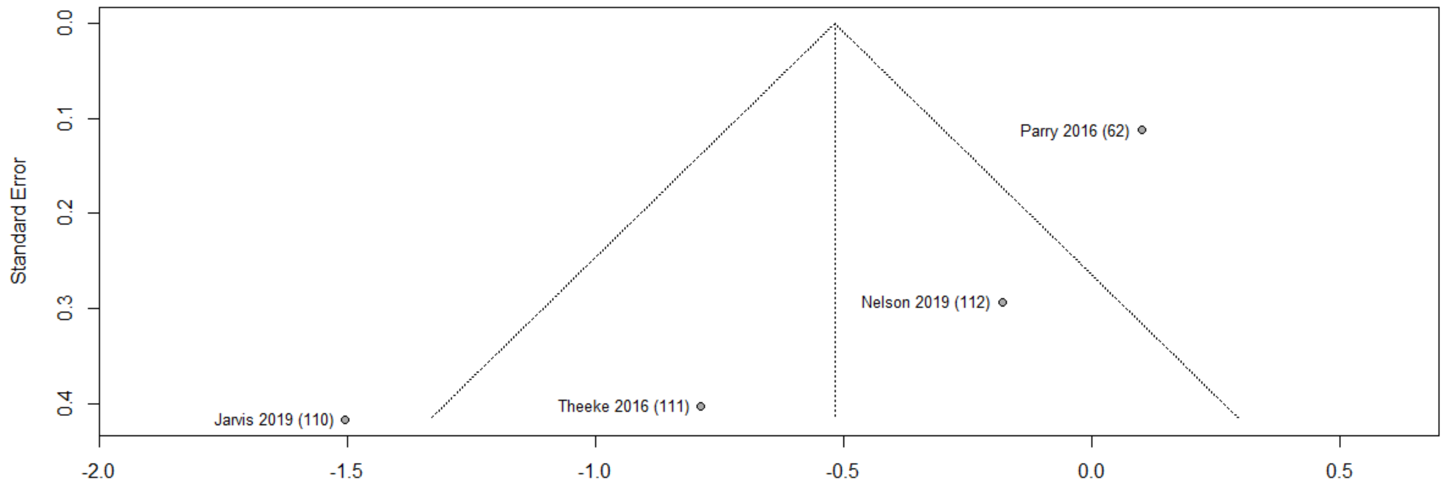

© 2022 Hoang P et al. *JAMA Network Open.*

a) Animal therapy: Community

Study	Intervention	N	Cohen's d	95% CI	Weight
Jessen 1996 (75)	Bird	40	0.25	[−0.37; 0.88]	50.9%
Robinson 2013 (70)	Interactive seal robot	34	−1.11	[−1.83; −0.39]	49.1%
**Random effects model**			**−0.41**	**[−1.75; 0.92]**	**100.0%**

Heterogeneity: $I^2 = 87\%$ [50%; 97%], $p < 0.01$

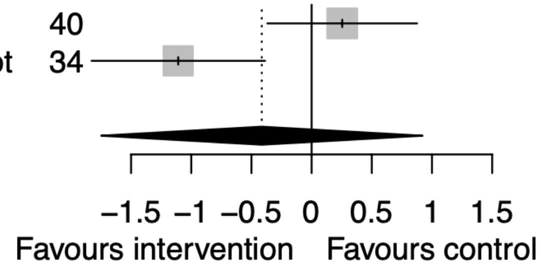

© 2022 Hoang P et al. *JAMA Network Open.*

b) Combined/Multi-component: Community

Study	Intervention	N	Cohen’s d	95% CI	Weight
Saito 2012 (77)	Group program	57	–0.73	[–1.29; –0.17]	67.3%
Gustafson 2019 (76)	Website and pedometer	25	–0.54	[–1.34; 0.26]	32.7%
**Random effects model**			**–0.67**	**[–1.13; –0.21]**	**100.0%**
Heterogeneity: I^2 = 0%, p = 0.70					

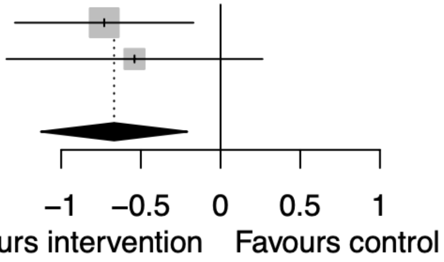

© 2022 Hoang P et al. *JAMA Network Open.*

c) Music therapy: Community

Study	Intervention	N	Cohen's d	95% CI	Weight
Johnson 2020 (92)	Music therapy (choir)	357	–0.34	[–0.55; –0.13]	100.0%

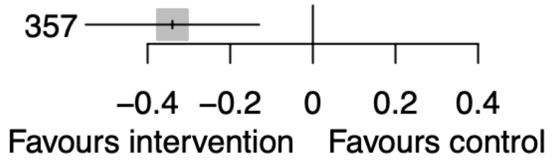

© 2022 Hoang P et al. *JAMA Network Open.*

d) Occupational therapy: Community

Study	Intervention	N	Cohen's d	95% CI	Weight
de Craen 2006 (93)	Occupational therapy	297	0.00	[–0.23; 0.23]	54.3%
Larsson 2016 (94)	Occupational therapy	28	–1.37	[–2.19; –0.55]	45.7%
**Random effects model**			**–0.63**	**[–1.96; 0.71]**	**100.0%**

Heterogeneity: $I^2 = 90\%$ [63%; 97%], $p < 0.01$

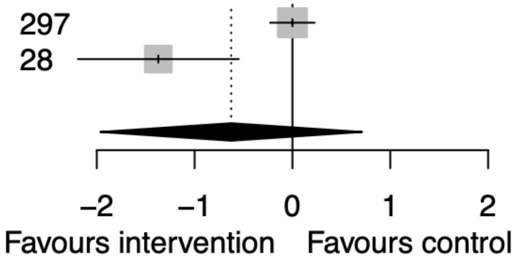

© 2022 Hoang P et al. *JAMA Network Open.*

e) Reminiscence therapy: Community

Study	Intervention	N	Cohen's d	95% CI	Weight
Moieni 2020 (97)	Reminiscence therapy	73	–0.70	[–1.17; –0.22]	100.0%

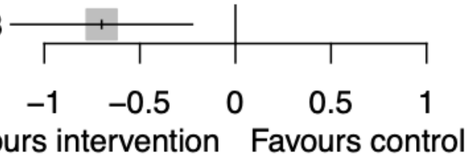

© 2022 Hoang P et al. *JAMA Network Open.*

f) Reminiscence therapy: LTC

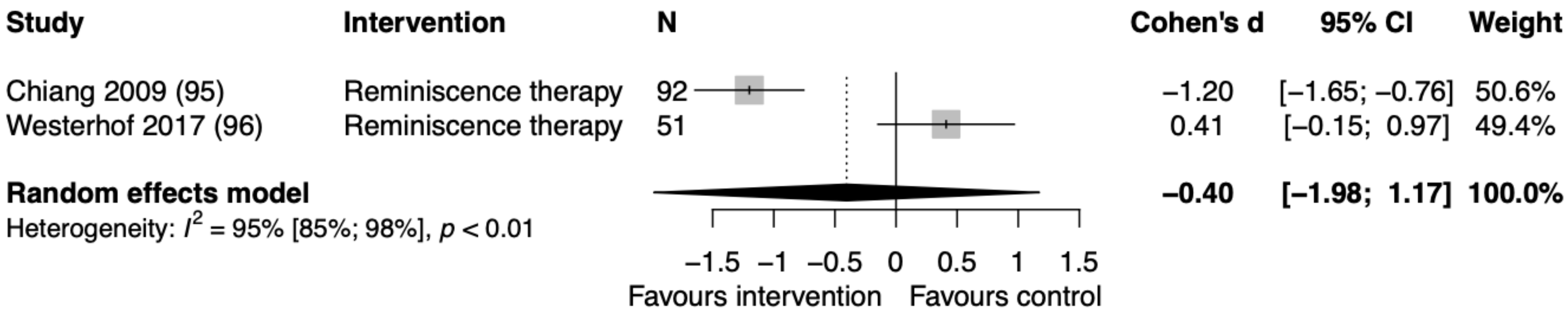

© 2022 Hoang P et al. *JAMA Network Open.*

eFigure 2: Risk of bias assessment of studies included in the systematic review.

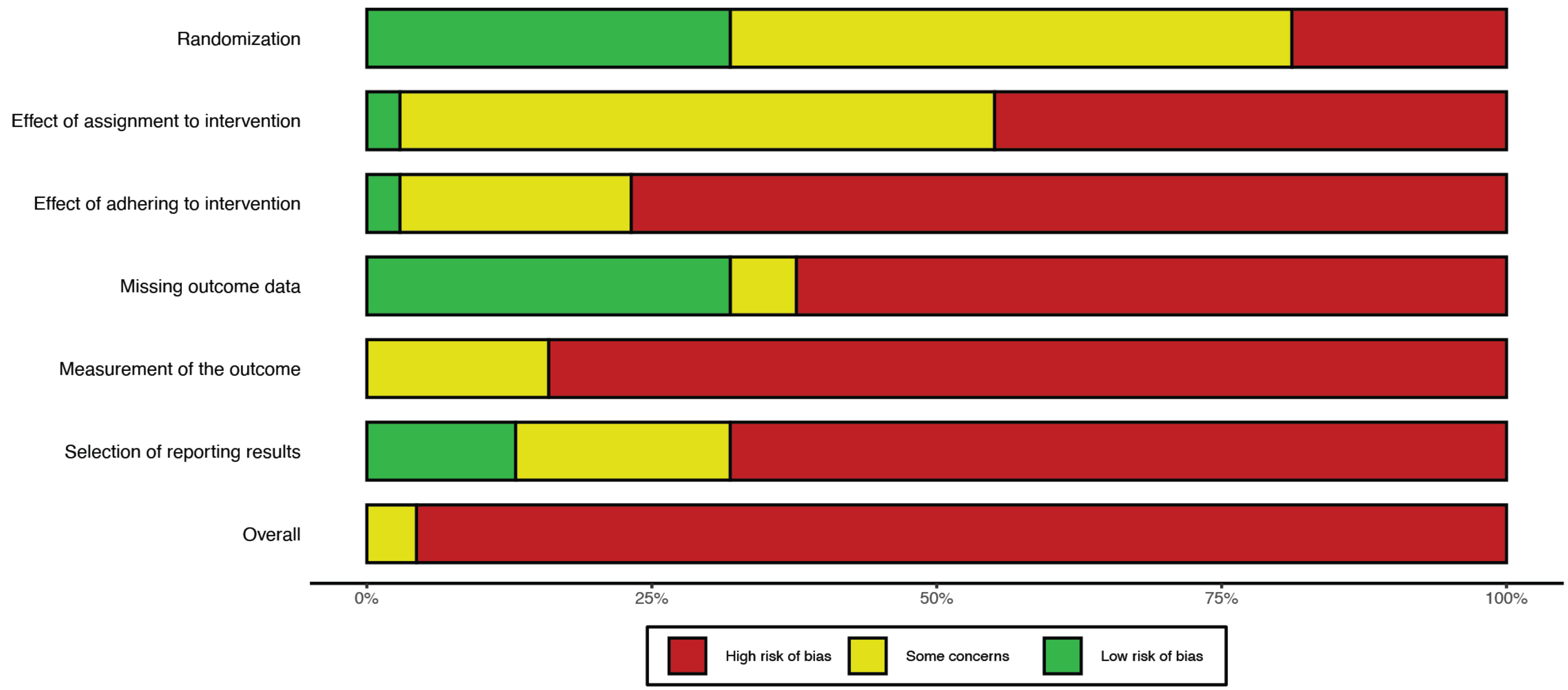

© 2022 Hoang P et al. *JAMA Network Open.*
